# Potential for Cell-Transplant Therapy with Human Neuronal Precursors to Treat Neuropathic Pain in Models of PNS and CNS Injury: Comparison of hNT2.17 and hNT2.19 Cell Lines

**DOI:** 10.1155/2012/356412

**Published:** 2012-04-24

**Authors:** Mary J. Eaton, Yerko Berrocal, Stacey Q. Wolfe

**Affiliations:** ^1^Miami VA Health System Center, D806C, 1201 NW 16th Street, Miami, FL 33199, USA; ^2^Department of Cellular Biology and Pharmacology, Herbert Wertheim College of Medicine, Florida International University, Miami, FL, USA; ^3^Department of Neurosurgery, Tripler Army Medical Center, 1 Jarrett White Road, Honolulu, HI 96859-5000, USA

## Abstract

Effective treatment of sensory neuropathies in peripheral neuropathies and spinal cord injury (SCI) is one of the most difficult problems in modern clinical practice. Cell therapy to release antinociceptive agents near the injured spinal cord is a logical next step in the development of treatment modalities. But few clinical trials, especially for chronic pain, have tested the potential of transplant of cells to treat chronic pain. Cell lines derived from the human neuronal NT2 cell line parentage, the hNT2.17 and hNT2.19 lines, which synthesize and release the neurotransmitters gamma-aminobutyric acid (GABA) and serotonin (5HT), respectively, have been used to evaluate the potential of cell-based release of antinociceptive agents near the lumbar dorsal (horn) spinal sensory cell centers to relieve neuropathic pain after PNS (partial nerve and diabetes-related injury) and CNS (spinal cord injury) damage in rat models. Both cell lines transplants potently and permanently reverse behavioral hypersensitivity without inducing tumors or other complications after grafting. Functioning as cellular minipumps for antinociception, human neuronal precursors, like these NT2-derived cell lines, would likely provide a useful adjuvant or replacement for current pharmacological treatments for neuropathic pain.

## 1. Introduction

Despite improvements [1] in surgical management, physical therapy, and the availability of pharmacological agents, with a variety of delivery systems, many patients following peripheral and central neural injuries continue to suffer from intractable chronic pain [2]. Although opioids are the most commonly used agent for the control of pain, only about 32% of patients receive any significant relief with long-term use [3], but this often leads to untoward effects associated with drug dependence, tolerance, tolerability, drug diversion, and other side effects [4], including opioid-induced neurotoxicity. Non-opioid medications can attenuate some types of neuropathic pain, but seldom remove completely the painful sensation [5]. Recent attempts at classification of neuropathic, nociceptive, and other pain, aided by an IASP Taskforce [6], have been of help to understand mechanisms and to improve and devise better treatments for chronic pain. But with the frequency of inadequate or failed clinical trials to advance treatment options for these problems, especially for chronic neuropathic pain [5], the development of translational cell therapies [7, 8], from stem cell [9, 10] or cell line sources [11], and use of newer animal models [12], is driving new interest in more sophisticated techniques for these problems [13–17].

Spinal cord injury (SCI) is a devastating clinical problem with injury severity directly related to not only motor paralysis, but also a large host of secondary complications [18] that challenge the injured person and their support network. The clinical presentation of chronic neuropathic pain following SCI is common but under-reported and has proven difficult to treat [19]. Neuropathic pain results from the abnormal processing of sensory input due to damage to the nervous system and onset of SCI pain is usually weeks to months after injury [20]. Few research studies have examined SCI pain [21], given the paucity of animal models for SCI pain, and so far none has shown any drug to be effective for a significant number of people. Some treatments, like implanted morphine pumps, work well but only temporarily. Pharmacological and surgical treatments are rarely successful, given the lack of understanding of mechanisms and focused, reliable interventions.

Painful peripheral neuropathies [13, 22] and diabetic peripheral neuropathy (DPN) are important clinical pain syndromes [23] with an estimated prevalence of about 5+ million in the USA. Presently, since there is little clear understanding of underlying mechanisms [24, 25], there are few effective long term treatments [26] for these conditions and therefore efforts are needed to develop and test novel therapeutic interventions.

Transplants of primary cultured cells near the dorsal horn of the spinal cord that release peptides and neurotransmitters have offered a new direction in the treatment of chronic pain. But, primary cells are difficult to obtain, non-homogeneous, and would require that each batch be tested before clinical use. Transplantation of immortalized cell lines genetically modified to release neuroactive antinociceptive peptides [27, 28], inhibitory neurotransmitters [29, 30] and neurotrophins [31] in chronic pain, and to upregulate inhibitory neurotransmitter synthesis offers a renewable source of cells [10, 11, 32, 33] that can act as cellular minipumps, are able to respond to the microenvironment of the spinal cord, and should reduce or eliminate side effects associated with the large doses of pharmacologic agents required for centrally-acting pain-reducing agents.

A human embryonal carcinoma cell line, NTera2cl.D/l (NT2), when treated with retinoic acid (RA), differentiates irreversibly into several morphologically and phenotypically distinct cell types, which include terminally differentiated postmitotic CNS neurons [34]. Successive replating of RA-treated hNT2 cells, in the presence of growth inhibitors, results in the isolation of purified human neurons [35] that have been extensively characterized and tested in vivo in a number of animal models of traumatic injury and neurodegenerative disease [36]. The potential application of hNT2 neurons in cell transplantation therapy for CNS disorders, without tumor formation in humans after transplants of differentiated cells [37], and their use as vehicles for delivering exogenous proteins into the human brain for gene therapy has been demonstrated [38, 39]. Such NT2 neurons have been used in Phase II clinical trials for the treatment of stroke [40] and have been approved by FDA for such trials [41].

Two major phenotypes present within the NT2 population are those which synthesize the inhibitory neurotransmitters gamma-aminobutyric acid (GABA) and serotonin (5HT) [42]. Taking advantage of rapid methods of proliferation and differentiation of the NT2 parent cell line in vitro, two subcloned novel human cell lines which are exclusively neuronal and of a specific neurotransmitter phenotype, the differentiated GABA hNT2.17 and 5HT hNT2.19 cell lines, have been studied over the past few years in a variety of animal models of neuropathic pain [10, 11, 43–47] for their ability to attenuate the loss of sensory and motor function after PNS and CNS injuries. Here, we summarize the conclusions derived from those studies, with additional data results from the transplant of these human cell lines in models of diabetic peripheral neuropathy (DPN) pain, unilateral chronic constriction injury (CCI) to the sciatic nerve, and excitotoxic SCI, with an interest towards describing the pros and cons involved in their further development as clinical tools to induce recovery-of-function and, especially, to treat neuropathic pain.

## 2. Materials and Methods

### 2.1. Development of the Human hNT2.17 and hNT2.19 Cell Lines

 Human neuronal cell lines were subcloned from the parental NTera2cl.D/l (NT2; hNT2) [34] cell line by serial dilution and analysis of multiple cell lines using a variety of immunohistochemical markers, including GABA and 5HT, to determine the differentiated neurotransmitter phenotype of the various cell lines. We took advantage of a rapid aggregation method [48] for retinoic acid (RA) treatment and differentiation into the human NT2-derived neuronal phenotype to select various cell lines, as reported previously [43, 47]. Although we derived a number of human hNT2 neurotransmitter cell lines by these methods, we have used the specific hNT2.17 and hNT2.19 cell lines for transplant in models of neuropathic pain in the peripheral (PNS) and central nervous system (CNS). The rapid aggregation method [48] for RA treatment and differentiation was also used for the preparation of cultures of differentiated hNT2.17 and hNT2.19 cells in vitro for characterization and transplant. Briefly, proliferating cultures of hNT2.17 or hNT2.19 cells were grown to near confluence at 37°C in proliferation medium: Dulbecco's Modified Eagle Medium/Ham's F12 (DMEM/F12, Gibco)/10% fetal bovine serum (FBS, HyClone, Logan, Utah)/2 mM L-glutamine (Gibco) freshly added/1% Pen-Strep (P.S.; Gibco) with an every 3rd day media change. When cells were near 100% confluent, they were replated to 100 mm Petri dish (VWR) in DMEM/high-glucose (HG)/10% FBS/10 *μ*M all-trans retinoic acid (RA) (Sigma)/15 mM HEPES, pH 8.0/2 mM L-glutamine/1%Pen-Strep, and continued for two weeks, with fresh media changed every 2 days. After removal with 0.5 mM EDTA, centrifugation and resuspension, cells were re-plated to 100 mm tissue culture dishes (Falcon) which had been coated with mouse laminin ((Biomedical Technologies, Stoughton, MA, USA; 20 *μ*g/mL in DPBS)/polyL-l-ysine (Sigma; 20 *μ*g/mL in PBS)). The cell cultures were then continued in DMEM/high-glucose (HG)/5% FBS/1% Pen-Strep (P.S.)/L-glutamine, 2 mM, at a pH of 7.4, for 9–24 hrs, before the addition of cytosine- D-arabinofuranoside (araC) (Sigma; 1 *μ*M), plus uridine (Sigma; 10 *μ*M), for non-neuronal growth inhibition. After seven days, cells were briefly exposed to warmed trypsin/0.5 mM EDTA, and adherent surface cells (hNT2 neurons) removed with DMEM/HG/5% FBS/P.S./L-glutamine, 2 mM, at a pH of 7.4. The cells were centrifuged, re-suspended, and re-plated on 60 mm tissue culture dishes (Falcon), which had been coated with mouse laminin ((Biomedical Technologies, Inc; 20 Φg/mL in DPBS)/poly-L-lysine (Sigma; 20 *μ*g/mL)) and continued in DMEMHG/5% FBS/P.S./L-glutamine, 2 mM at a pH of 7.4 at 37°C for two weeks before transplant, with media change every 2-3 days. Three cell lines, the hNT2.17, hNT2.19, and negative control hNT2.6, were isolated and developed for use in transplant studies.

### 2.2. Immunohistochemistry of the hNT2.17 and hNT2.19 Cell Lines In Vitro

Monoclonal antibody anti-bromodeoxyuridine (BrdU; #347580; dilution 1 : 10) was purchased from Becton-Dickson, San Jose, CA, USA. The polyclonal antibody anti-5HT (ab10385-50; dilution 1/100 (in vitro)) was purchased from Abcam Inc, Cambridge, MA, USA. Monoclonal antibody anti-beta-tubulin III (TuJ1, MO15013; dilution 1 : 100) was purchased from Neuromics, Edina, MN, USA. The polyclonal antibody anti-GABA (dilution 1 : 100) was purchased from Protos Biotech Corporation, New York, NY. Monoclonal antibody anti-NuMA (dilution 1 : 20 (in vivo)) was purchased from Calbiochem, San Diego, CA, USA. The hNT2.17 and Hnt-19 cells, after two weeks of RA treatment and mitotic inhibitors, were re-plated to differentiate in 8-well laminin/poly-L-lysine coated Permanox slides, and differentiation continued for 1-2 weeks before immunostaining. The cells were then fixed for 10 min at 4°C with 4% paraformaldehyde and 0.1% glutaraldehyde in 0.1 M phosphate buffer, pH 7.4. All immunohistochemistry experiments included the use of a negative control, substitution of specific primary antibody with species IgG, to insure that positive signal was specific for the antigen. For the anti-BrdU immunostaining: after fixation and rinsing in PBS, pH 7.4 at room temperature, hNT2.17 or hNT2.19 cells were incubated with 2 N HCl for 20 min at room temperature, rinsed x3 with PBS, incubated with borate buffer (pH 8.5)/0.01 M boric acid/0.5 M Na borate (1 : 1) for 15 min at room temperature, rinsed for three times with PBS, and then permeabilized for 30 min at room temperature with blocking buffer before incubation with the primary anti-BrdU antibody. For all other in vitro immunostaining experiments: after fixation and rinsing in PBS, pH 7.4 at room temperature, fixed hNT2.17 or hNT2-19 cells were permeabilized for 30 min at room temperature with 0.5% Triton X-100 in PBS in the presence of 5% normal goat serum (the blocking buffer), before the addition of the individual primary antibody, usually overnight at 4°C. The staining was completed by incubation with the specific anti-species IgG secondary conjugated to Alexa Fluor 488 Green (dilution 1 : 100), purchased from Molecular Probe, Eugene, OR, USA, for two hours at room temperature. After staining, slides were cover-slipped using Vectashield mounting medium with DAPI (Vector Laboratories, Burlingame, CA, USA). Photo images were taken with a Zeiss microscope (Axioplan II Metamorphosis program). All staining experiments were independently repeated at least x3, to insure that micrographs are representative.

### 2.3. Immunohistochemistry of the hNT2.17 and hNT2.19 Cell Lines In Vivo

#### 2.3.1. Fixation

Spinal cords were fixed for examination of cell graft survival, GABA, and nuclear matrix antigen (NuMA) staining (for hNT2.17 grafts) or survival, 5HT, and TuJ1 antigen stain (for hNT2.19) eight weeks after DPN, QUIS, or contusive SCI. Transcardial perfusion with Lana's fixative (4% paraformaldehyde and 0.1% glutaraldehyde in PBS) was performed. Rats were euthanized for tissue fixation by a combination of pentobarbital overdose (i.p. injection, 12 mg/100 g) and exsanguination. Once the appropriate level of anesthesia was reached (i.e., no corneal or withdrawal reflexes), rats were transcardially perfused with aldehydes. After perfusion, the spinal cords, including transplant, were removed and histologically processed. After removal from the vertebral column, cords were stored in fix for 12 h at 4°C. These cords were cryoprotected by equilibration in 30% sucrose and PBS overnight at 4°C and then frozen and stored at −80°C. Cords were embedded in Shandon-1 Embedding Matrix (Thermo Electron Corp; Waltham, Ma, USA) and sagittally cut in sequential 20 *μ*m sections with a Cryostat (Leica CM3050 S Cryostat, Micro Optics of Florida Inc; Davie, Fl, USA). They were collected on non-coated slides (micro Slides, Snowcoat X-tra, Surgipath; Richmond, Il, USA). The slides were stored in a −20°C freezer and removed for defrosting before the immunostaining procedures. Every second section was stained for the anti-human marker NuMA or GABA (for hNT2.17 grafts) or anti-human TuJ1 and 5HT (for hNT2.19 grafts) and dehydrated, cleared, and mounted in Cytoseal 60 (Richard-Allan Scientific (Thermo Electron Corp)) after antibody staining. Processed slides were observed and photographed with a Zeiss Axioplan2 research microscope.

#### 2.3.2. GABA Staining in hNT2.17 Grafts

Methods for staining lumbar spinal cord sections for GABA have been adapted from methods described elsewhere [49]. Sections were incubated with the primary antibody anti-GABA (1 : 500; Protide Pharmaceuticals, Inc.) with 0.4% Triton-X-100 in 0.1 M PBS and 10% NGS overnight at 4°C, followed a one hour incubation at room temperature with the secondary antibody solution, biotinylated guinea pig raised in goat (Vector) in 0.4% Triton-X-100 in 0.1 M PBS and 10% normal goat serum (NGS), a Peroxidase ABC reporter in 0.1 M PBS (Vector) and “VIP” substrate (Vector). Some sections were stained in the absence of primary antibody, and served as the negative controls.

#### 2.3.3. 5HT Staining in hNT2.19 Grafts

Methods for staining lumbar spinal cord sections for 5HT and grafted hNT2-derived cell lines have been adapted from methods described elsewhere [46]. Sections were incubated with the primary antibody anti-5HT (1 : 100) with 0.4% Triton-X-100 in 0.1 M PBS and 10% NGS overnight at 4°C, followed a one hour incubation at room temperature with the secondary antibody solution, biotinylated anti-rabbit IgG (H+L), made in goat (Vector; 1/200) in 0.4% Triton-X-100 in 0.1 M PBS and 10% normal goat serum (NGS), a Peroxidase ABC reporter in 0.1 M PBS (Vector) and “VIP” substrate (Vector). Some sections were stained in the absence of primary antibody and served as the negative controls.

#### 2.3.4. NuMA Staining in hNT2.17 Grafts

Methods for staining spinal cord sections for the human nuclear matrix antigen (NuMA) to identify hNT2 neurons after grafting have previously been described [50]. The sections were washed with 0.1 M PBS pH 7.4 and permeabilized with 0.4% Triton-X-100 in 0.1 M PBS, 10% normal goat serum (NGS) and 3% poly-D-lysine (Sigma) for one hour. The sections were then incubated overnight at 4EC in the primary anti-NuMA antibody (EMD Bioscience; 10 mg/mL DPBS), and the permeabilizing solution, followed by a one-hour incubation at room temperature with the secondary antibody solution, biotinylated mouse raised in goat (Vector), a Peroxidase ABC reporter in 0.1 M PBS (Vector) and “VIP” substrate (Vector). Some sections were stained in the absence of primary antibody and served as the negative controls.

#### 2.3.5. TuJ1 Staining in hNT2.19 Grafts

Modified methods for staining spinal cord sections for the human neuron-specific class III beta-tubulin (TuJ1) to identify grafted hNT2.19 neurons after grafting have previously been described [51]. The sections were washed with 0.1 M PBS pH 7.4 and permeabilized with 0.4% Triton-X-100 in 0.1 M PBS, 10% normal goat serum (NGS) for one hour. The sections were then incubated overnight at 4°C in the primary anti-TuJ1 antibody (1 : 100 DPBS), and the permeabilizing solution, followed by a one-hour incubation at room temperature with the secondary antibody solution, biotinylated mouse IgG raised in goat (Vector; 1 : 200), a Peroxidase ABC reporter in 0.1 M PBS (Vector) and “VIP” substrate (Vector). Some sections were stained in the absence of primary antibody and served as the negative controls.

### 2.4. HPLC of Neurotransmitters in hNT2 Cell Lines In Vitro

#### 2.4.1. GABA in hNT2.17 Cells In Vitro

In order to examine the GABA content and release in differentiated hNT2.17 cells, cells were differentiated for 2 wks at 37°C after plating in 35 mm laminin/poly-L-lysinecoated 6well plates. Cell numbers were determined in sister wells by trypan blue exclusion and counting. Either GABA content (in cells) or GABA secretion or release (into the media) was examined by HPLC to determine the content or basal or stimulated level of GABA secretion or release, respectively, into the media. For GABA content: cells were collected into 1.5 mL centrifuge tube (in distilled water), cells broken by two 10 sec bursts of ultrasound, tube contents centrifuged at 4°C, and the supernatant collected for HPLC. Similar cell culture samples were also incubated with either normal K+ (2.95 mM) KrebsRinger buffer or high K+ (100 mM) buffer for 15 min at 37°C, and the media collected to determine the levels of GABA released into the media by membrane depolarization. The media samples were kept on ice and immediately analyzed by HPLC. An o-phthaladehyde (OPA) pre-column derivatization and reverse-phase isocratic liquid chromatography with electrochemical detection, as described previously [52] were used. The HPLC system consisted of a solvent-delivery pump (Waters 510 Pump); an autosampler (Waters 717 plus Autosampler) and an electrochemical detector (ESA Coulochem II; Electrode: ESA Model 5011 Analytic Cell; Guard Cell: Model 5020). Elution was carried out at room temperature with a reversed-phase column (3 *μ*m, C18, 80 × 4.6, HR80, ESA) and a mobile phase of 0.1 M sodium acetate (pH.5)-acetonitrile (73 : 27, v/v) at a flow rate of 0.6 mL/min. To an OPA solution (2 mg of o-phthaldialdehyde (OPA) in 0.2 mL methanol), first 0.8 mL of 0.1 M borax buffer (pH 10.4) and 5 *μ*L of 2-mercaptoethanol were added. Four minutes before the injection on the column, 1 : 4 volumes of the OPA reagent and sample were mixed and incubated at room temperature by autosampler. After injection, the GABA peak appearance time was about 5 min in 27% Ace mobile phase. 

#### 2.4.2. 5HT in hNT2.19 Cells In Vitro

In order to examine the 5HT content, secretion, and release in differentiated hNT2.19, cells were differentiated for 2 wks at 37°C after plating in 35 mm laminin/poly-L-lysine-coated 6-well plates. Cell numbers were determined in sister wells by trypan blue exclusion and counting. Either 5HT content (in cells) or 5HT secretion or release (into the media) was examined by HPLC to determine the content or basal or stimulated level of 5HT release into the media. For 5HT content, cells were collected into 1.5 mL centrifuge tube (in distilled water), cells broken by lysis with 0.05 N PCA (perchloric acid), tube contents centrifuged at 4°C, and supernatant collected for HPLC. Similar cell culture samples were also incubated with either normal K+ (2.95 mM) Krebs-Ringer buffer or high K+ (100 mM) buffer for 15 min at 37°C and the media collected to determine the levels of 5HT released into the media by membrane depolarization. The media samples were kept on ice and immediately analyzed by HPLC. The HPLC system consisted of a solvent-delivery pump (Waters 510 Pump), an autosampler (Waters 717 plus Autosampler), and an electrochemical detector (ESA Coulochem II); Electrode: ESA Microdialysis Cell 5014A (DC CH1: 150 mV, DC CH2: 300 mV, 500 mA); Guard Cell Model 5020 (GC 350 mV). Elution was carried out at room temperature with a reversed-phase column (C18, 5 M, 150-3, BetaBasic-18, Thermo) and MDTM mobile phase (ESA Inc. 70–1332); it consisted of 75 mM of NaH2PO4, 1.7 mM of C_8_H_17_O_3_SNa, 100 *μ*L/L of TEA, 25 M of EDTA, 10% acetonitrile, pH 3.0 adjusted by H_3_PO_4_ at a flow rate of 0.6 mL/min. Ordinarily the 5HT appeared at about 7.5 min.

### 2.5. Surgeries and Cell Transplant

#### 2.5.1. Unilateral Chronic Constriction Injury (CCI) of the Sciatic Nerve and hNT2 or hNT2.17 Cell Transplant

The surgery to produce CCI was first described by Bennett and Xie [53]. This model of injury has been used by our laboratories and many others to test the effects of cell transplants to relieve pain-related behaviors [54]. Under ketamine/xylazine anesthesia, the right common sciatic nerve was exposed at the level of the middle thigh by blunt dissection through the biceps femoris. Proximal to the nerve's trifurcation, a 5–7 mm of nerve was freed of adhering tissue and 4 ligatures (4.0 chromic gut) were tied loosely around it with 1 mm spacing. Care was taken to tie the ligatures so that the diameter of the nerve was barely constricted, so that vigorous tactile allodynia (TA) and thermal hyperalgesia (TH) behaviors lasted at least 10 wks after CCI. The incision was closed in layers and the entire surgery was repeated, minus the ligatures, on the left side to create a sham-operated nerve. At two weeks following the CCI, and following a partial laminectomy with a small puncture of the dura, either viable or nonviable parental hNT2 or hNT2.17 cells (predifferentiated 2 wks; 10^6^ cells/injection) were injected into the subarachnoid space of the lumbar dorsal spinal cord, by a dorsal/caudal entry into the dural puncture a few millimeters with a small length of polyethylene (PE-10) tubing containing the cells, at spinal segment L1. For both CCI and transplantation, animals were anesthetized with a mixture of ketamine, xylazine, and acepromazine, 0.65 mL/Kg. Animals were allowed to recover at 37°C for 12 hrs, after which time they were returned to the animal care facility, and housed 2/cage with rat chow and water ad lib on a 12/12 hr light/dark cycle.

All surgical interventions, pre- and postsurgical animal care, and euthanasia were performed in accordance with the Laboratory Animal Welfare Act, Guide for the Care and Use of Laboratory Animals (National Institutes of Health; Department of Health, Education and Welfare, Pub. No. 78-23, Revised 1978) and the guidelines provided by the Animal Care and Use Committees of the Department of Veterans Affairs Medical Center and the University of Miami, both in Miami, Fl, USA. All behavioral testing was performed under blinded conditions to eliminate experimental bias; the data were analyzed and un-blinded by the statistician at the end of the experiment.

#### 2.5.2. Streptozotocin-Induced Diabetic Peripheral Neuropathy and hNT2.17 Cell Transplant

Animals were administered an IV injection (in the tail vein) of STZ (50 mg/Kg) dissolved in 0.9% (w/v) physiological saline, made to approximately 10 mg/25 *μ*L. Immediately before, about 3 days and periodically post-STZ injection all rats had their blood glucose measured with a glucometer, utilizing <1 *μ*L of blood removed by tail prick. Rats with blood glucose >250 mg/dL were considered diabetic and were assigned to one of the control groups or to receive cell transplants. In all animals, except those not to receive cell grafts (at 5 days after STZ) and the development and measure of sensory behaviors, animals received lumbar subarachnoid grafts of human (nonviable or viable hNT2.17) cells. A partial T13L1 laminectomy was performed and 10^6^ cells/injection in 5–10 *μ*L sterile Hank's buffered saline was injected into the subarachnoid space of the lumbar dorsal spinal cord, by a dorsal/caudal entry into the dural puncture a few millimeters with a small length of small length of polyethylene (PE-10) tubing containing the cells, at spinal segment L1. Following transplantation, the exposed surface of the spinal cord was covered with dura-film, the overlying musculature was sutured and the wound closed with wound clips. Animals were allowed to recover on warming blankets (under the cage corner) for 12 hrs, after which time they were returned to the animal care facility and housed 2/cage with rat chow and water ad lib on a 12/12 hr light/dark cycle.

All surgical interventions, pre- and postsurgical animal care, and euthanasia were performed in accordance with the Laboratory Animal Welfare Act, Guide for the Care and Use of Laboratory Animals (National Institutes of Health; Department of Health, Education and Welfare, Pub. No. 78-23, Revised 1978) and the guidelines provided by the Animal Care and Use Committees of the Department of Veterans Affairs Medical Center and the University of Miami, both in Miami, Fl, USA. All behavioral testing was performed under blinded conditions to eliminate experimental bias; the data were analyzed and unblinded by the statistician at the end of the experiment.

#### 2.5.3. Excitotoxic Spinal Cord Injury (QUIS) and hNT2.17 or hNT2.19 Cell Transplant

The spinal QUIS injury procedure has been previously described [55]. To produce this excitotoxic injury, quisqualic acid (QUIS; non-synthetic, Sigma), a glutamate receptor agonist, was administered in sufficient concentrations (125 mM) to cause neuronal cell loss and demyelination. The animals were anesthetized with a mixture of ketamine, xylazine, and acepromazine (0.65 mL/kg). A laminectomy was performed between T12-L1. The rat was then placed in a stereotaxic frame and the dura and arachnoid incised. Using a micropipette attached to a Hamilton syringe, the QUIS was unilaterally injected into the dorsal horn, 1000 *μ*m below the surface of the cord, in three separate injections 500 *μ*m apart. Each injection was 0.4 *μ*L in volume for a total of 1.2 *μ*L. Anatomically, the injection was located midway between the central vein and dorsal root entry zone, just lateral to the posterior columns. On pathologic examination, these unilateral injections were centered in the gray matter between the spinal laminae IV-VI. A small piece of sterile dura-film was placed over the dura (to protect the spinal cord and facilitate reopening the dura for transplantation) and the fascia and skin were closed. Other than the anesthesia, no additional perioperative analgesics were given. The animal was recovered at 37°C for 12 hours and then returned to the animal care facility. Two weeks later, an aliquot of one million cells was prepared immediately prior to each transplant to assure near 100% viability at the beginning of the experiment; grafting was within 30 min of cell preparation. Nonviable hNT2.17 cells were prepared by initially re-suspending one million cells in sterile water, centrifugation, checking viability, then resuspension in CMF-HBSS for transplant.

 The animals to be transplanted, one day after showing a vigorous response to behavioral testing, were anesthetized with a mixture of ketamine, xylazine, and acepromazine (0.65 mL/kg). The previous laminectomy site (T12-L1) was exposed. A small dural and arachnoidal incision was made and a 2-3 mm segment of polyethylene (PE-10) tubing, connected to a micropipette, inserted through the durotomy in a caudal direction. The one million cells (either hNT2.17 or hNT2.19) were injected into the intrathecal space at spinal segment L1-L3 and the fascia and skin closed. Again, no additional analgesia was used. The animals were allowed to recover at 37°C for 12 hrs, after which time they were returned to the animal care. All rats, including those not provided cell transplants, received immunosuppressive therapy with cyclosporine A (CsA; 10 mg/Kg), injected i.p., which began one day before cell transplant, or 13 days after QUIS or saline injection, and continued daily for 14 days.

All surgical interventions, pre- and postsurgical animal care, and euthanasia were performed in accordance with the Laboratory Animal Welfare Act, Guide for the Care and Use of Laboratory Animals (National Institutes of Health; Department of Health, Education and Welfare, Pub. No. 78-23, Revised 1978) and the guidelines provided by the Animal Care and Use Committees of the Department of Veterans Affairs Medical Center and the University of Miami, both in Miami, Fl, USA. All behavioral testing was performed under blinded conditions to eliminate experimental bias; the data were analyzed and un-blinded by the statistician at the end of the experiment.

#### 2.5.4. Severe Contusive Spinal Cord Injury and hNT2.17 and hNT2.19 Cell Transplant

Contusion injury was induced by the weight-drop device developed at New York University [56]. Animals were anesthetized using an i.p. injection of a mixture of ketamine (35 mg/Kg) and xylazine (5 mg/Kg), all 0.65 mL/Kg, and then placed on a surgical table on a heating pad (37°C) with pedal and eye blink reflexes assessed for deep anesthesia before beginning procedures. The back region was shaved and aseptically prepared with betadine. Lacrilube ophthalmic ointment (Allergan Pharmaceuticals, Irvine, CA, USA) was applied to the eyes to prevent drying and Bicillin (0.02 mL/100 mg body weight, 300 U/mL; J. Buck, Inc., Owings Mills, MO, USA) administered intramuscularly. Following anesthesia, a vertical incision was made along the thoracic vertebra and the superficial muscle and skin retracted. A laminectomy performed at thoracic vertebra T7 exposed the dorsal surface of the spinal cord underneath (T8) without disrupting the dura mater. Stabilization clamps were placed around the vertebrae at T6 and T12 to support the column during impact. The exposed spinal cord was severely injured by dropping a 10.0 g rod from a height of 25.0 mm. The contusion impact velocity and compression were monitored to guarantee consistency-of-injury between animals. After injury, the muscles were sutured in layers and the skin closed with absorbable sutures (Ethicon Inc). The rats were allowed to recover in a warmed cage with water and food easily accessible. Bicillin (0.02 mL/100 mg body weight, 300 U/mL, i.m.) was administered 2, 4, and 6 d after the contusion injury. The rats were maintained for 8 wks after injury, including gentle twice daily manual bladder expression to prevent the development of cystitis. For cell transplant 2 weeks after QUIS SCI, viability and cell counts were assessed by trypan blue exclusion, and the cells were suspended in 10–20 *μ*L of Ca_2+_-Mg_2+_ free Hank's buffered saline solution (CMF-HBSS). An aliquot of one million cells (10^6^ cells/injection) was prepared immediately prior to each transplant to assure near 100% viability at the beginning of the experiment; grafting was within 30 min of cell preparation. The animals to be transplanted, one day after showing a vigorous response to behavioral testing, were anesthetized with a mixture of ketamine, xylazine, and acepromazine (0.65 mL/kg). For subarachnoid grafts, the previous laminectomy site (T7) was exposed and a small dural and arachnoidal incision was made and a 2-3 mm segment of polyethylene (PE-10) tubing, connected to a micropipette, inserted through the durotomy in a caudal direction. The one million cells (hNT2.17 or hNT2.19 cells) were injected into the intrathecal space at spinal segment L1–L3 and the fascia and skin closed. Again, no additional analgesia was used. The animals were allowed to recover at 37°C for 12 hrs, after which time they were returned to the animal care facility. All rats, including those not provided cell transplants, received immunosuppressive therapy with CsA, injected i.p., which began one day before cell transplant and continued daily for 13 days, unless otherwise noted.

All surgical interventions, pre- and postsurgical animal care, and euthanasia were performed in accordance with the Laboratory Animal Welfare Act, Guide for the Care and Use of Laboratory Animals (National Institutes of Health; Department of Health, Education and Welfare, Pub. No. 78-23, Revised 1978) and the guidelines provided by the Animal Care and Use Committees of the Department of Veterans Affairs Medical Center and the University of Miami, both in Miami, Fl, USA. All behavioral testing was performed under blinded conditions to eliminate experimental bias; the data were analyzed and un-blinded by the statistician at the end of the experiment.

### 2.6. Sensory Behavioral Testing

#### 2.6.1. Tactile Allodynia

Mechanical allodynia, the occurrence of foot withdrawal in response to normally innocuous mechanical stimuli, was tested using an automated, electronic von Frey anesthesiometer (IITC, Inc) [57]. Animals were placed in a plexiglass box with an elevated mesh floor. After the animal was acclimated for 5 min, the device tip was applied perpendicular to the midplantar area of each hindpaw and depressed slowly until the animal withdrew the paw from pressure. The value, in grams, was recorded for each of the 3 trials. A single trial of stimuli consisted of three to four applications of the von Frey tip within a 10-second period, to ensure a consistent response. The values obtained for each hindpaw were averaged and the SEM calculated. The animals were tested 3 times, one week apart, for 2-3 wks prior to the injury (baseline), and then weekly for the duration of the experiment. In order to provide a robust baseline value for comparison purposes, all baseline data was averaged to a mean baseline based on the three baseline tests.

#### 2.6.2. Thermal Hyperalgesia

Methods for testing thermal hyperalgesia with a Hargreaves device have been described elsewhere [58]. Animals were placed in a clear plexiglass box on an elevated plexiglass floor. Animals were allowed to acclimate for approximately 5 min. A constant intensity, radiant heat source was aimed at the midplantar area of the hind paws. The time, in seconds, from initial heat source activation until paw withdrawal, was recorded. Five minutes were allowed between assessments. Three to four latency measurements for each paw were recorded and the mean and standard error of the mean (SEM) calculated for each hindpaw. Animals were tested 3 times, one week apart, for 2 wks prior to the injury (baseline) and then weekly for the duration of the experiment. In order to provide a robust baseline value for comparison purposes, baseline data was averaged to a mean baseline based on the three baseline tests.

### 2.7. Motor Behavior Testing

#### 2.7.1. Open-Field Motor Behaviors (BBB)

Two weeks prior to the injury, open-field locomotor functions of all animals were assessed using the Basso, Beattie, and Bresnahan (BBB) locomotor rating scale [59]. Behavioral assessments were then performed on days 1 and 7 following the injury and weekly thereafter. The BBB score was used to study the functional recovery stages following the injury, by categorizing the rat hindlimb movements, trunk position and stability, coordination, stepping, and paw placement and tail position. Rats were placed in a small, shallow, empty children's swimming pool and allowed to move freely for 60 mins of exercise, during which their motor behaviors were observed and scored according to the BBB scale. All observations were made by at least two independent observers, who were unaware of the extent or nature of the injury. The animals were rated on a scale of 0 to 21.

#### 2.7.2. Fine Motor Behaviors (BBB Subscores)

The BBB scale alone may not reflect changes in the finer details of locomotion (e.g., paw positioning, toe clearance) [60]. Therefore, since paw position and toe clearance are routinely documented once animals are able to step consistently, BBB analysis was supplemented by subscoring the fine details of locomotion at the plateau of the recovery phase (at weekly time points before and before injury). A subscore, 0–5, was given to each hindlimb based on paw rotation and toe clearance of the hindlimb. Subscores were assigned as follows: paw position, 0.5 rotation at initial contact and liftoff, 1.5 rotation at initial contact or liftoff and parallel at initial contact/liftoff, 2.5 parallel at initial contact and liftoff; and toe clearance, 0.5 no clearance, 1.5 occasional clearance (50% of the time), 2.5, frequent clearance (51–95%), 3.5, consistent (95%) clearance. The cumulative scores of each hindlimb were summed to yield a single score (maximum score of 10/rat). All observations were made by at least two observers, who were unaware of the nature or extent of injury.

### 2.8. Statistical Analysis

Statistical analyses were performed with PASW 17.0 for Windows. To determine differences between the groups and between time points, one-way analysis of variances (ANOVAs) and paired Student' *t*-tests were used. All *t*-tests were two-tailed, and Bonferroni correction to adjust for multiple comparisons were used. A *P* value of  .05 or less was considered statistically significant.

## 3. Results

### 3.1. Morphology and Phenotype of hNT2.17 and hNT2.19 Cells In Vitro

**Figure 1:**
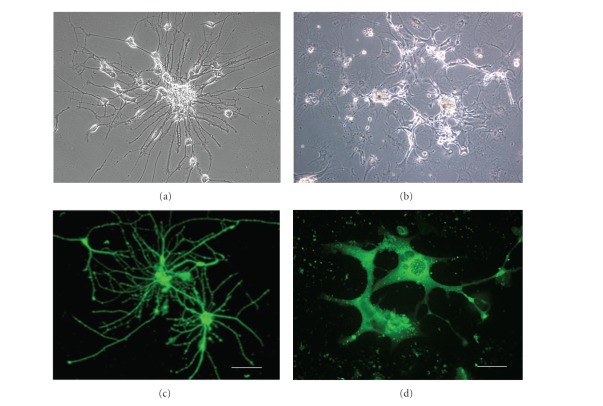
Human neuronal hNT2.17 GABA and hNT2.19 5HT cell lines in vitro. The GABA hNT2.17 (a, c) and 5HT hNT2.19 (b, d) cell lines were subcloned by serial dilution and treated for 2 wk with retinoic acid and mitotic inhibitors. They were further differentiated for 2 wk before either phase microscopy (a, b) or stained with anti-GABA (c) or -5HT (d) antibodies, respectively. Magnification: bar = 50 nm, (a–c); 25 nm (d).

It is essential that differentiated cells useful for clinical transplant be homogeneous and with a well-defined phenotype, since the functionality of any clinical transplant source depends on easily identifiable cell-synthesized agents. Both the hNT2.17 [43, 61] and hNT2.19 [47] cell lines have been characterized previously. As seen in Figure 1, differentiated hNT2.17 and hNT2.19 cells are distinctly different in morphology and phenotype. The GABAergic hNT2.17 have small nuclei, with long neurites (Figure 1(a)), and stain intensely for GABA (Figure 1(c)), while the 5HT NT2.19 cells have very large nuclei and are generally multi- or bipolar, with short neurites (Figure 1(b)), and stain brightly for 5HT (Figure 1(d)) after differentiation in vitro. Both cell lines are exclusively neuronal following differentiation [43, 47, 61], expressing a variety of human and neuronal markers, including neuron-specific enolase and neurofilament proteins, with the hNT2.17 cell line able to synthesize high molecular-weight neurofilament protein (NFH) slightly later than the hNT2.19 cell line in culture, but both appear to express a mature neuronal phenotype, including neurite extensions and bouton-like structures, that co-localize other important neurotransmitters, such as glycine, especially in the hNT2.17 cell line. Also, in the hNT2.17 cells both the GAT3 plasmolemma GABA re-uptake and vesicular inhibitory amino acid transporter (VIAAT) are abundant at 2 weeks of differentiation [43], another marker of a mature neuron. Important to any clinical use for transplant, both cell lines can be kept for long periods (>30 days), and both form increasingly dense neurite mats, with aggregate balls of cell bodies, over long periods in culture. 

### 3.2. Both Cell Lines Cease Proliferation with Differentiation In Vitro

**Figure 2:**
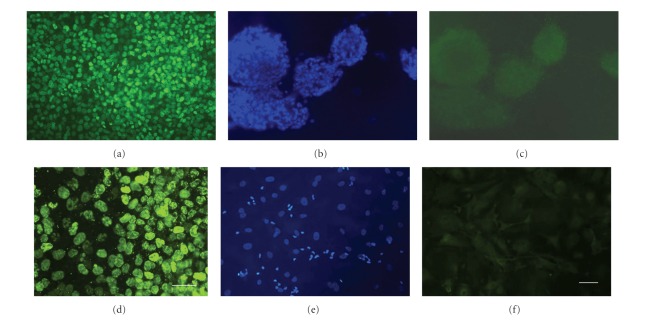
hNT2.17 and hNT2.19 cells are nontumorigenic: BrdU stain. hNT2.17 (a–c) and hNT2.19 (d–f) cells were exposed to 1 *μ*M bromodeoxyuridine (BrdU) (a, d) during 3 days of proliferation or (b, c, e, f) for 1 week during differentiation in vitro. Proliferating cells (a, d) incorporate abundant BrdU during proliferation. Viable differentiated cells (b, e) were labeled with DAPI (4′6-diamidino-2-phenylindal-2HCl) stain, while the same field (c, f) of differentiated cells did not incorporate any BrdU during differentiation. After 2 weeks of treatment with retinoic acid (RA) and mitotic inhibitors, hNT2.17 and hNT2.19 cells cease dividing and differentiation proceeds without further cell division. Magnification bar = 20 nm (d); 30 nm (a, b, c, e, f).

The parental tumorigenic NT2 cell line is known to change its phenotype to nontumorigenic after differentiation with RA in vitro [35], downregulating key tumor genes after RA [62, 63], allowing it to be transplanted into the central nervous system (CNS) [38, 64] and used safely in human studies [41, 65–67]. Here, the hNT2.17 and hNT2.19 cell lines switch from a proliferating to a nonproliferating phenotype after RA exposure and treatment with mitotic inhibitors. Both hNT2.17 [43] and hNT2.19 [47] cell lines downregulate their expression of the tumor-proteins TGF-*α* and FGF-4 with RA exposure and differentiation in vitro. Bromodeoxyuridine (BrdU) immunostaining has also has been used as a marker for proliferating cells in vitro [68] and in vivo [69], since dividing cells incorporate BrdU-labeled uridine into newly made deoxyribonucleic acid (DNA).  Figure 2 illustrates the loss of the BrdU signal with differentiation in culture in each cell line. The hNT2.17 and hNT2.19 cells were exposed to 1 *μ*M BrdU in vitro during either proliferation or differentiation before anti-BrdU immunostaining. Following 3 days of proliferation in the presence of BrdU, the BrdU signal was intense and found in all the dividing cells hNT2.17 (Figure 2(a)) and hNT2.19 (Figure 2(d)) cells. After 1 week of BrdU exposure during the first week of differentiation, hNT2.17 (Figure 2(b)) and hNT2.19 (Figure 2(e)) cells remained viable, as evidenced by DAPI staining. The same field of differentiated hNT2.17 (Figure 2(c)) and hNT2.19 (Figure 2(f)) cells showed no BrdU signal, demonstrating the inability to incorporate BrdU during differentiation in these two cell lines. 

### 3.3. The hN2.17 and hNT2.19 Cell Lines Synthesize and Secrete the Neurotransmitters GABA and 5HT, Respectively, In Vitro

**Figure 3:**
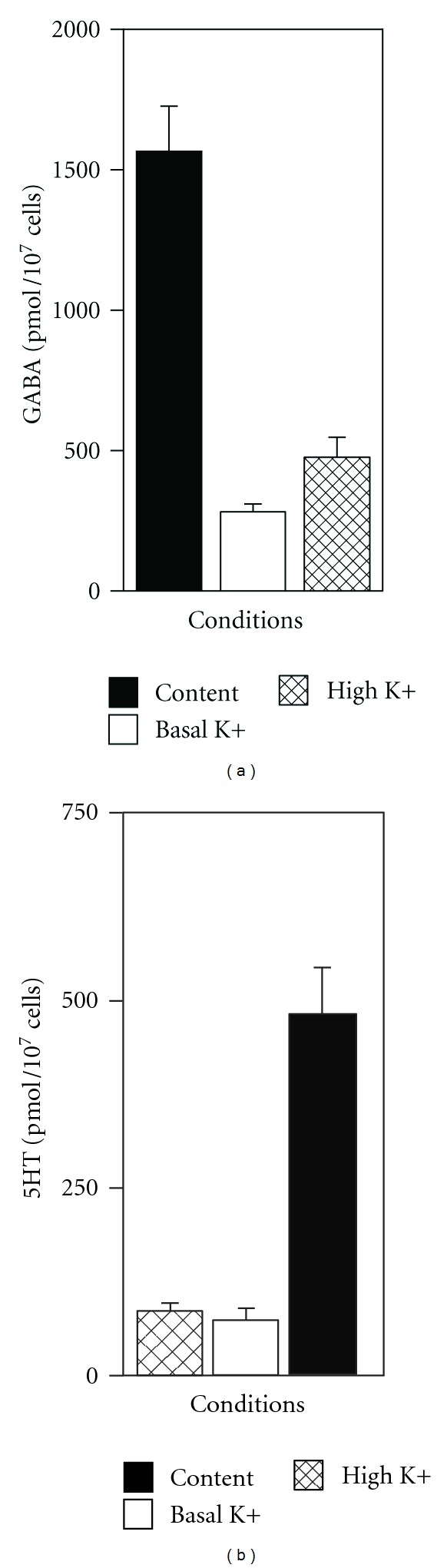
HPLC of GABA and serotonin in hNT2.17 and hNT2.19 cells. The hNT2.17 (a) and hNT2.19 (b) cell lines were differentiated, after RA and mitotic inhibitor treatment, for two weeks in 6-well substrate-coated plates before cell lysis and examination of cell content for GABA (a) or 5HT (b) by HPLC methods. For GABA or 5HT secretion (basal) and release (stimulated), sister cultures of the hNT2.17 or hNT2.19 cells were differentiated for two weeks before cells were exposed to basal (2.95 mM) or high (100 mM) concentrations of KCl for potassium (K+)-stimulated secretion/release for GABA or 5HT. Data represent the mean + SEM from 3-4 samples from >4 independent experiments for each neurotransmitter.

Since the neurotransmitters GABA and 5HT each play a major role in antinociception with nervous system injury, stable human neuronal cell lines with these specific neurotransmitter phenotypes, and able to secrete the GABA or 5HT directly into the cellular environment, near the spinal cord, are a good choice for transplant in models of neuropathic pain. The hNT2.17 GABA and hNT2.19 5HT cell lines were differentiated for 2 weeks in vitro before HPLC analysis of GABA in hNT2.17 (Figure 3(a)) or 5HT in hNT2.19 (Figure 3(b)) content, basal secretion in the presence of basal KCl (2.95 mmol/L), and stimulated release in the presence of high KCl (100 mmol/L) in the medium. The hNT2.17 cell line was able to synthesize significant amounts of the GABA neurotransmitter, matching the immunohistochemical staining patterns seen above. GABA content (Figure 3(a)) was 1567.88 (mean) pmoles per 10 million cells. The hNT2.17 cell line also demonstrated significant GABA release under basal or potassium-stimulated conditions at the time point during differentiation when these cells were transplanted in the QUIS SCI pain model [43]. GABA release under basal (mean of 281.95 pmoles per 10 million cells) or stimulated KCl conditions (mean of 471.16 pmoles per 10 million cells) during a period of 15 minutes was able to account for about 18% and more than 30%, respectively, of the total GABA content in the cell cultures. Although not shown here, glycine content, secretion, or release was approximately 10 times higher than that for GABA in the hNT2.17 [43]. Very different results are seen for the hNT2.19 cell line, which does not contain GABA, but instead synthesizes, secretes, and releases 5HT [47]. The hNT2.19 cell line synthesized significant amounts of the 5HT neurotransmitter, again matching the immunohistochemical staining patterns seen above. 5HT content (Figure 3(b)) was 485.13 (mean) pmoles per 10 million cells. The hNT2.19 cell line also demonstrated significant 5HT release under basal or potassium-stimulated conditions at the time point during differentiation when these cells were transplanted in the severe contusive SCI pain model [47]. 5HT release under basal (mean of 73.38 pmoles per 10 million cells) or stimulated KCl conditions (mean of 85.64 pmoles per 10 million cells) during a period of 15 minutes was able to account for about 15% and more than 17%, respectively, of the total 5HT content in the cell cultures. 

### 3.4. Recovery of Sensory Function in Peripheral Models of Neuropathic Pain with Transplant of GABA hNT2.17 Cells

Two common models [12] of pain-like sensations of peripheral origin and neuropathy have been used to examine the effects on behavioral hypersensitivity and transplants of hNT2-derived cell lines, especially the GABA hNT2.17 cell line: unilateral chronic constriction injury to the sciatic nerve (CCI) [30, 70] and diabetic peripheral neuropathy (DPN) pain with injection of streptozotocin (STZ) [71, 72]. Use of both models has revealed some interesting data concerning the inhibitory neurotransmitter GABA cell line hNT2.17, since a cell therapy approach to influence the GABA inhibitory system [11, 13, 30, 32, 73] has a long history, and similar pharmacologic intervention [74–77] is the most commonly used approach in these models.

#### 3.4.1. Chronic Constriction Injury (CCI) and Transplant of hNT2.17 Cells

**Figure 4:**
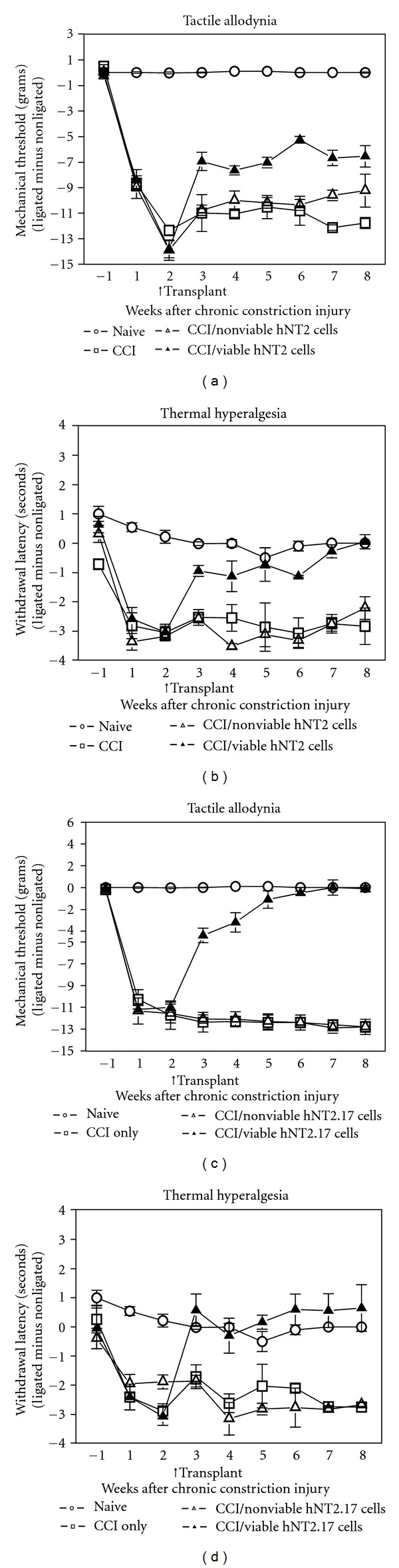
Sensory behaviors after initiation of peripheral CCI pain and intrathecal transplant of parental hNT2 and hNT2.17 cells. For tactile allodynia after CCI and transplant of parental (a) hNT2 cells, adult female rats were either left unoperated, underwent CCI, or transplanted with nonviable hNT2 or viable hNT2 cells two weeks following CCI, one day following behavioral testing. Nonviable cells were prepared by suspension of the cells in water, centrifugation, and resuspension in buffer before transplant. All rats received 10 mg/Kg i.p. CsA at the time points corresponding to one day before and 13 days after cell transplant (daily injections). Animals were tested for hindpaw withdrawal to a graded series of von Frey hairs once every week for one week before and eight weeks following CCI and before and after transplants. Only animals that demonstrated tactile allodynia two weeks after CCI were transplanted. The data reported are the mean ± SEM of the difference scores for ligated paw minus the sham-operated paw of 14 animals in each group. The results with viable hNT2 cell transplants differed significantly from the CCI or nonviable graft conditions at each time point. *P* < 0.001. For thermal hyperalgesia after CCI and transplant of parental (b) hNT2 cells, animals were tested for hindpaw withdrawal once every week for one week before and eight weeks following CCI and before and after transplants. Only animals that demonstrated thermal hyperalgesia 2 weeks after CCI were transplanted. The data reported are the mean ± SEM of the difference values for ligated paw minus the sham-operated paw of 14 animals in each group. The viable hNT2 transplants differed significantly from the CCI and nonviable graft condition at each time point. *P* < 0.001. For tactile allodynia after CCI and transplant of GABA (c) hNT2.17 cells, adult female rats were either left unoperated, underwent CCI, or transplanted with nonviable hNT2.17 or viable hNT2.17 cells two weeks following CCI, one day following behavioral testing. Animals were tested for hindpaw withdrawal once every week for one week before and eight weeks following CCI and before and after transplants. Only animals that demonstrated tactile allodynia two weeks after CCI were transplanted. The data reported are the mean ± SEM of the difference scores for ligated paw minus the sham-operated paw of 14 animals in each group. The viable hNT2.17 cell transplants differed significantly from the CCI or nonviable graft conditions at each time point. *P* < 0.001. For thermal hyperalgesia after CCI and transplant of (d) hNT2.17 cells, animals were tested for hindpaw withdrawal once every week for one week before and eight weeks following CCI and before and after transplants. Only animals that demonstrated thermal hyperalgesia 2 weeks after CCI were transplanted. The data reported are the mean ± SEM of the difference values for ligated paw minus the sham-operated paw of 14 animals in each group. The viable hNT2.17 transplants differed significantly from the CCI and nonviable graft condition at each time point. *P* < 0.001.

Both the hNT2.17 and hNT2.19 cell lines were subcloned from the parental NT2 cell line [35], but the parental hNT2 cell line has never been examined for it's potential to affect neuropathic pain in the CCI model. A comparison of the sensory effects in the CCI model by subarachnoid lumbar grafts of either the parental hNT2 (Figures 4(a) and 4(b)) or the GABA hNT2.17 cells (Figures 4(c) and 4(d)) is illustrated in Figure 4. When viable hNT2 grafts are placed two weeks after the CCI, and tactile allodynia (TA) (a) is examined, the maximum recovery-of-function is seen at six weeks, but never achieves more than about 40% of normal tactile responses by the end of the experiment (42 days). Both CCI alone and grafts of nonviable hNT2 cells had no effect on sensory recovery, and viable hNT2 cell grafts induced an improvement in TA and TH over injury alone or nonviable graft placement. When, the experiment is repeated with the same number of grafted cells placed again two weeks after CCI, where the grafted cells are either the viable or nonviable subcloned GABA hNT2.17 cells (c), recovery of sensory function is also seen within one week after cell transplant, but achieves about 60% of normal tactile responses at 21 days and 100% recovery by seven weeks. Again, grafts of nonviable hNT2.17 cells or CCI only had no effect on the development of tactile allodynia. The hNT2.17 grafts were far more effective than similar grafts of the parental hNT2 cells. Similar results are seen in results of an examination of TH responses with the grafts of either the parental hNT2 cells (b) or the subcloned GABA hNT2.17 cells (d), but the maximum effect of GABA hNT2.17 is immediate (100% recovery of normal TH responses), seen at 21 days. In comparison, graft of parental hNT2 cells does not achieve 100% recovery-of-thermal function until 8 weeks, with only about 65% recovery at 21 days. Also, like TA responses, there is no effect on TH recovery by either CCI or grafts of nonviable hNT2 or hNT2.17 cells. Overall, the viable hN2.17 grafts were far more effective on recovery of normal sensory function, compared to transplant of viable parental hNT2 cells. 

#### 3.4.2. Diabetic Peripheral Neuropathy (DPN) Pain and Transplant of hNT2.17 Cells

**Figure 5:**
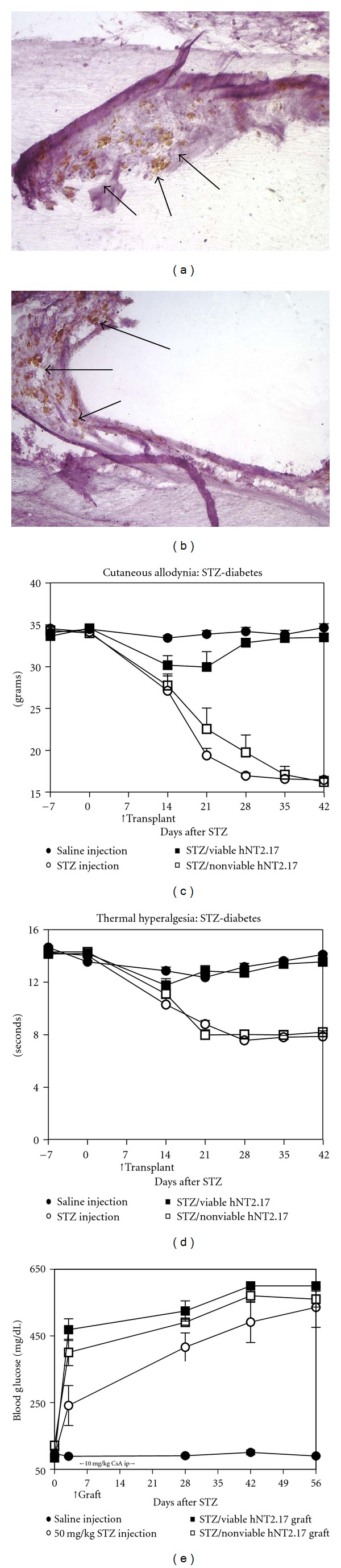
Sensory behaviors after diabetic peripheral neuropathy (DPN) and intrathecal transplant of hNT2.17 cells. The hNT2.17 cells were transplanted into the lumbar subarachnoid space 5 days after a STZ injection (50 mg/Kg, i.v.) and cords fixed at 42 days after STZ and transplants. (a) Cords were examined for graft survival with the human NuMA marker (arrows) and adjacent sections (b) stained with the antibody marker for GABA (arrows). Many surviving hNT2.17 cells, expressing both NuMA and GABA could be found on the pial surface (arrows), especially over the dorsal lumbar spinal cord. For cell transplant and sensory behavior evaluation, nonviable cells were prepared by suspension of the cells in water, centrifugation, and resuspension in buffer before transplant. All rats received 10 mg/Kg i.p. CsA at the time points corresponding to one day before and 13 days after cell transplant (daily injections). Tactile allodynia behaviors (c) were examined for both a baseline period before STZ injection and for 42 days following STZ. Two groups of STZ-injected rats (*n* = 6) were transplanted with either 1 × 10^6^ hNT2.17 viable or nonviable hNT2.17 cells at 5 days following STZ, a time when the behavioral hypersensitivity to nonnoxious tactile stimulation was already apparent in the rats. In these three groups (nontransplanted and transplanted), both hindpaws develop (pooled data) increased hypersensitivity, but only the rats with viable hNT2.17 cell grafts recover permanent tactile responses. Saline injected rats never develop tactile allodynia and serve as positive controls. The data (mean + SEM) is from six rats in each group. For thermal hyperalgesia (d), the same rats were examined on alternate days for responses to noxious thermal stimulation and like tactile allodynia, thermal hyperalgesia was apparent immediately before the transplant time, 5 days after STZ. In these three groups (non-transplanted and transplanted), both hindpaws develop (pooled data) increased hypersensitivity, but only the rats with viable hNT2.17 cell grafts recover permanent normal thermal responses. Saline injected rats never develop thermal hyperalgesia and serve as positive controls. The data (mean + SEM) is from six rats in each group. (e) All rats were examined for blood glucose levels before and after STZ and transplant of hNT2.17 cells. Data are the mean ± SEM from >six rats in each group. No treatment had an effect on the vigorous increase in blood glucose levels induced by 50 mg/Kg STZ injection; only the saline injected animals showed no increase in blood glucose.

DPN pain as an animal model of pain of peripheral origin takes advantage of the toxic, destructive effect of STZ, delivered either i.p. or i.v. into the tail vein, to the pancreas [71]. In our hands, an i.v. route of administration of the drug is the most dependable, and induces vigorous TA and TH within 3 days after injection, at a dose of 50 mg/Kg. When viable hNT2.17 cells are transplanted in the lumbar subarachnoid space at 5 days after STZ injection (Figure 5(a)), and the transplant site examined at 42 days with either anti-NUMA (a) or anti-GABA (b) immunohistochemistry, many viable, GABAergic hN2.17 cells can been seen in the subarachnoid space (arrows), with a 14-day course of CsA immunosuppression, peritransplant time. STZ injection induces permanent, increasing, and vigorous TA (c) and TH (d) behaviors within 3 days of drug administration, where only grafts of viable GABA hNT2.17 cells, placed 5 days after STZ, are able to recover permanent normal sensory responses (100% by 35 days in TA; 100% by 21 days in TH behaviors), compared to STZ alone or STZ/nonviable grafts. However, this sensory recovery-of-function did not affect the great increase in blood glucose levels, seen in the presence or absence of grafts, with STZ injection (e).

### 3.5. Recovery-of-Function in Central (SCI) Models of Neuropathic Pain and Motor Dysfunction with Transplant of hNT2.17 and hNT2.19 Cells

Two well-described models of SCI, with accompanying loss of sensory and motor function have been used to examine the effects of transplants of hNT2-derived cell lines, namely the quisqualic (QUIS) chemical lesion, induced by the unilateral spinal injection of quisqualic acid [78] and the severe contusive SCI model [56], induced by weight-drop spinal contusion (NYU impactor). Even with significant differences in these injuries and outcomes [12], both induce permanent TA and TH behaviors, with the QUIS model also inducing permanent > excessive = grooming behaviors in the ipsilateral hindpaw. Permanent motor dysfunction (bilateral) is induced by severe contusive SCI. Perhaps the QUIS model may be characterized as a pure sensory model of central pain, since no motor paralysis is induced, while the severe contusive SCI induces both motor and sensory loss-of-function. Both offer certain advantages and disadvantages, not the least of which might be the greatly increased animal survival in the QUIS model, compared to severe contusive SCI, with no loss of bladder function, no paralysis, as long as “excessive grooming” behaviors are not too severe. And only a severe contusion (>25 mm weight-drop) SCI can be used for an examination of sensory dysfunction, since <25 mm weight-drop (e.g., 12.5 or 6 mm) does not provide consistent TA and TH [79], since injury severity and sensory/motor outcomes depend on weight-drop distance with the NYU impactor device [80]. Less severe contusion SCI models are more often used in a variety of cell therapy approaches to recovery-of-function, for example, Schwann cell grafts for sensory/motor recover and regeneration [81].

#### 3.5.1. Graft Sites after SCI and Transplant of hNT2.17 or hNT2.19 Cells

**Figure 6:**
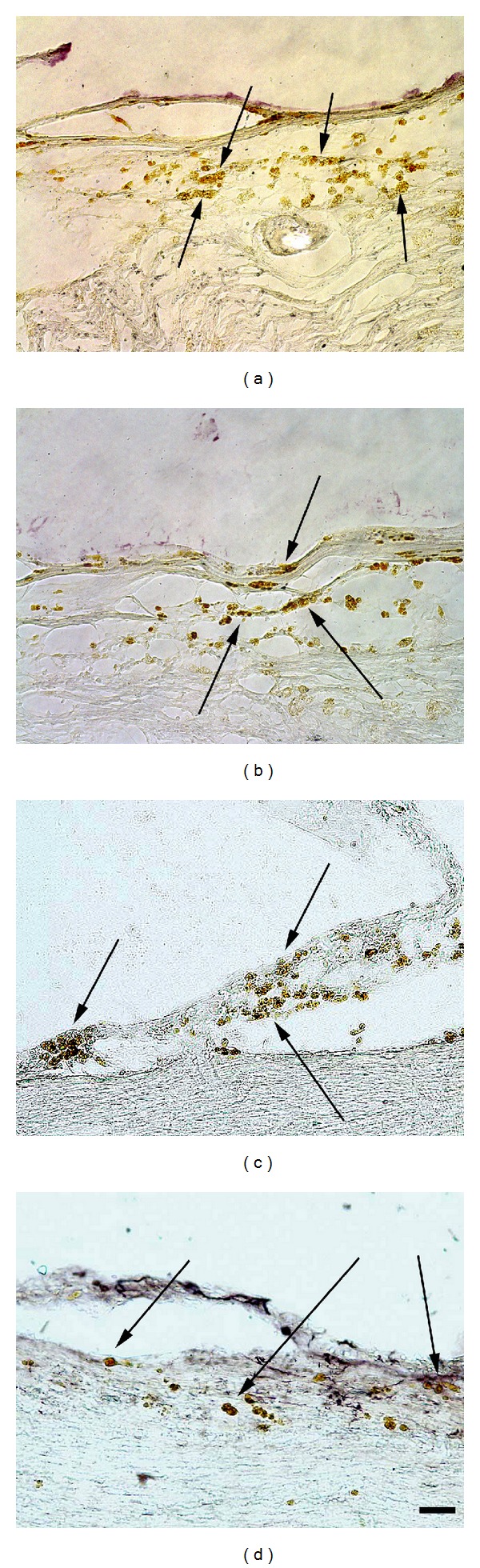
Comparison of graft sites of hNT2.17 and hNT2.19 in the QUIS and severe contusive-SCI models, respectively, at 6 weeks after cell transplant. (a) Sagittal section of anti-GABA-immunostained QUIS + hNT2.17 transplant lumbar spinal cord 6 weeks after grafting. Easily detectible hNT2.17 cells stain for GABA (arrows) on the pial membranes. (b) Sagittal section of anti-NuMA-immunostained QUIS + hNT2.17 transplant lumbar spinal cord 6 weeks after grafting. Easily detectible hNT2.17 cells stain for NuMA (arrows) on the pial membranes in adjacent sections. The hNT2.19 were alternately injected into the subarachnoid space two weeks after severe contusive SCI. Cell graft sites were co-localized with 5HT (c) and the human-specific marker TuJ1(d) (neuron-specific class III *β*-tubulin). There are many surviving hNT2.19 (d) grafted cells visible on the pial surface, which stain for TuJ1 (arrows) at the end of the experiment, 56 days after SCI and about 6 weeks after cell transplant. Adjacent sections with the same grafted hNT2.19 (c) are labeled for 5HT (arrows). Magnification bar = 20 *μ*m.

Graft sites after the transplant of human cells can easily be located with anti-human cell immunohistochemistry for either the markers NuMA or TuJ1, both used to find the graft of parental hNT2 cells in previous studies [51]. Both markers have also been used previously to locate grafted hNT2.17 [43] and hNT2.19 [47] cells in various models of pain. Here we illustrate anti-NuMA (Figures 6(a) and 6(b)) and -TuJ1 (Figures 6(c) and 6(d)) stained hNT2.17 (a, b) and hNT2.19 (c, d) grafts in the QUIS and contusive SCI models, respectively. Both cell line grafts survive well and maintain their neurotransmitter phenotypes at least 6 weeks after transplant in these two models of SCI pain, excitotoxic (QUIS), and severe contusive SCI. 

#### 3.5.2. Direct Comparison of Behavioral Sensitivity Following Grafts of Either hNT2.17 Or hNT2.19 Cells in the Same Model of QUIS SCI

**Figure 7:**
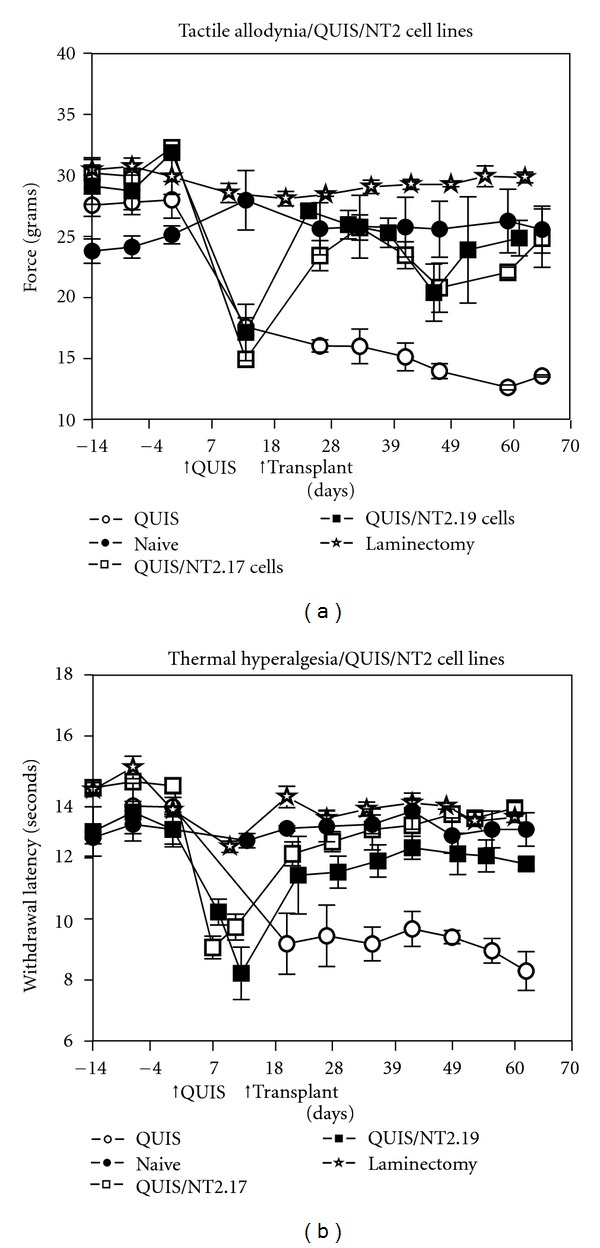
Comparison of sensory recovery after transplant of hNT2.17 or hNT2.19 cell lines in the same QUIS model of SCI. Rats were spinally injected with QUIS in a rat model of SCI and chronic pain. All animals in the study received CsA (10 mg/Kg) 1 day before and for 13 days after the two week time-point when some animals were injected with either the hNT2.17 or hNT2.19 cells. Animals were either left untreated (naïve), injected with QUIS alone or laminectomy only or QUIS plus hNT2.17 or hNT2.19 cells (10^6^ cells/injection) into the lumbar subarachnoid space at two weeks after QUIS. Animals were tested before the SCI (baseline) and once a week following QUIS and treatments for hypersensitivity to tactile (a) or thermal (b) stimuli in hindpaws below the SCI. QUIS injury negatively affected hindpaw responses bilaterally, but the ipsilateral hindpaw is most affected by the injection of quisqualic acid (shown here). Neither hindpaw recovers normal tactile or thermal responses after QUIS alone by 60 days after the injection. Both ipsilateral and contralateral hindpaws recovered near-normal sensory responses to tactile and thermal stimuli after grafting the GABAergic hNT2.17 or serotonergic hNT2.19, compared to the QUIS injury alone. Data represent the mean value ± SEM (*n* = 4–6 animals in each group) at each time point before and 63 days after QUIS.

The hNT2.17 and hNT2.19 cell lines were directly compared for their ability to attenuate TA and TH behaviors in the same central model of neuropathic pain, the excitotoxic SCI model, illustrated (Figures 7(a) and 7(b)). In these experiments, a laminectomy alone injury was compared to QUIS SCI alone, with naive animals providing baseline data. There was no significant difference with TA (a) or TH (b) in the data from the naive or laminectomy alone animals throughout the course of the experiment (63 days after QUIS). QUIS alone SCI induced a vigorous TA (a) and TH (b) behavioral response as soon as 14 days after the injury, that persisted and worsened over the course of the experiment and was different at every time point from the naive or laminectomy data. For TA behaviors (a), lumbar subarachnoid transplant at 14 days after QUIS of either hNT2.17 or hNT2.19 cells (10^6^ cells/injection) immediately attenuated the TA behaviors (to near normal) at 21 days. The attenuation persisted, with little difference between the cell line grafts, until 42 days, when the attenuation briefly dropped until 56 days, when attenuation began to recover to near normal levels by the end of the experiment, where data was indistinguishable from naive animals. For TH behaviors (b), transplant of either cell line had an immediate effect on TH, attenuating the behaviors to near normal levels at 21 days, an effect which persisted throughout the 63-day experiment. Overall, there was little difference on attenuation of TA and TH behaviors between transplants of the GABA hNT2.17 or 5HT hNT2.19 cell lines in the same QUIS model of neuropathic pain; both achieving near normal recovery-of-sensory function immediately after transplant, an effect that persisted through 63 days.

#### 3.5.3. Percent Comparison of Sensory Recovery in the QUIS and Severe Contusive SCI Models with Transplant of hNT2.17 and hNT2.19 Cells

**Figure 8:**
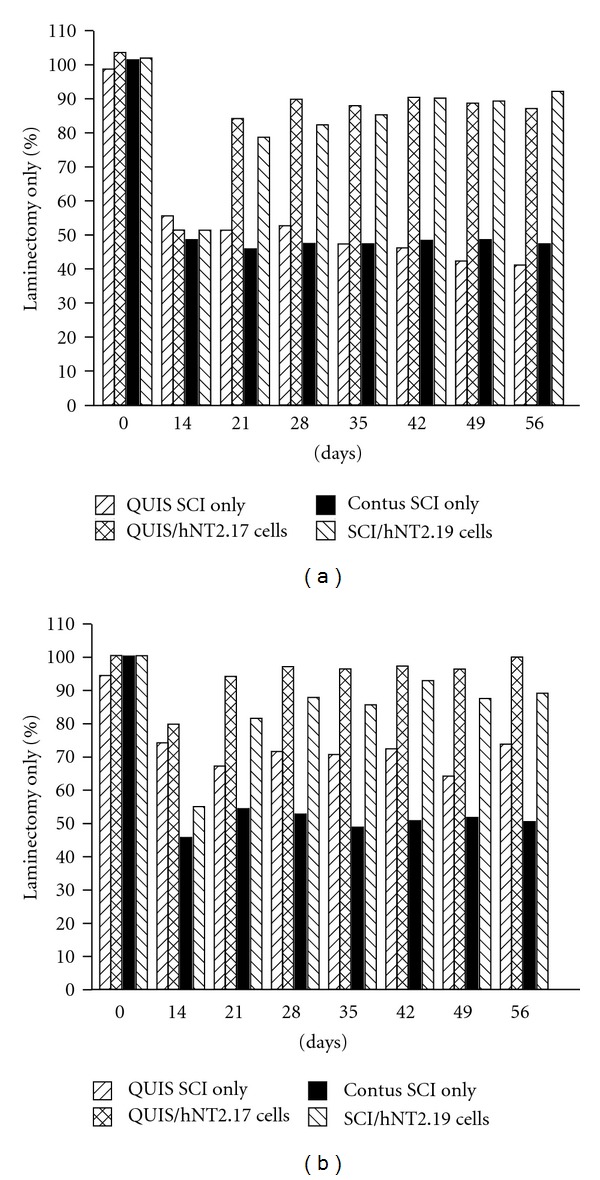
Percent comparison of sensory behaviors after QUIS SCI or severe contusive SCI and with transplant of hNT2.17 or hNT2.19 cells in vivo—type of hNT2-derived cell graft. Rats were either injured with QUIS injection SCI or with a weight-drop device (NYU impactor, 25 mm; severe contusive SCI) in rat models of SCI and chronic behavioral hypersensitivity. All animals in the study received CsA (10 mg/Kg) 1 day before and for 13 days after the two week time-point (14 days) when some animals were injected with hNT2.17 (QUIS model) or hNT2.19 cells (severe contusion SCI). Animals either received one of the SCIs alone, laminectomy alone, or one of the SCIs plus hNT2.17 or hNT2.19 cells (10^6^ cells/injection) into the subarachnoid space at two weeks after SCI. Animals were tested before the SCI (baseline), and once a week following SCI and treatments for hypersensitivity to tactile (a) or thermal (b) stimuli in hindpaws below the SCI. Data are the mean of percent of laminectomy control data in each model, where SCI injury alone negatively affected hindpaw responses and laminectomy alone had no effect. Data represent the mean value (*n* = 4–9 animals in each group) at each time point before and 56 days after SCI. In these different models, the hNT2.17 and hNT2.19 and cell grafts potently and comparably attenuated tactile allodynia (a) and thermal hyperalgesia (b) induced by either SCI. Recovery of normal behaviors was near-complete by experiment's end with graft of either hNT2.17 or hNT2.19 cells.

Although the two central models of SCI pain used here cannot easily be directly compared, each cell line was examined for its sensory behavioral effects, hNT2.17 cells in the QUIS model and hNT2.19 cells in the contusive SCI model, and the data normalized to percent of laminectomy control data results for each experiment (Figures 8(a) and 8(b)). For TA behaviors (a), QUIS SCI alone induced a 44.4% increase in TA (55.6% of laminectomy) by 14 days after injection. In comparison, contusive SCI induced a 51.2% increase in TA (48.8% of laminectomy) by 14 days following the contusion. Both lumbar subarachnoid grafts were placed immediately following measure of this data, at 14 days after SCI: hNT2.17 (10^6^ cells/injection) in QUIS-injured animals and hNT2.19 (10^6^ cells/injection) in contusive SCI animals that demonstrated the same increases in TA behaviors. By 21 days (7 days after transplant in both models), when QUIS SCI alone had induced a 48.6% increase in TA (51.4% of laminectomy), the addition of hNT2.17 grafts to the QUIS SCI had attenuated the TA to 84.3% of laminectomy. Similarly, at 21 days, the addition of hNT2.19 grafts to the contusive SCI had attenuated the TA behaviors to 78.8% of laminectomy, compared to a 54% increase (46% of laminectomy) with the contusive SCI alone. These comparisons continue throughout the 56-day experiment: the QUIS SCI increases in TA, with the highest allodynia reached at 56 days, with 59.4% (40.6% of laminectomy), when the addition of hNT2.17 grafts had decreased the TA to 89.6% of laminectomy control. Similarly, the contusive SCI alone had the worst TA at 56 days, 47.6% of laminectomy, while the addition of hNT2.19 grafts to the SCI showed near normal recovery, 92.2% of laminectomy. For TH behaviors (b), at 14 days the QUIS SCI induced a 25.6% increase (74.4% of laminectomy), compared to a 44.9% increase (55.1% of laminectomy) for contusive SCI. By 21 days, the addition of hNT2.17 grafts to the QUIS SCI had improved the TH to 94.4% of laminectomy, compared to only 67.4% of laminectomy for QUIS SCI. By the end of the experiment (56 days), the addition of hNT2.17 grafts had induced a 100% recovery, compared to only 74% for QUIS SCI alone. Similar results were found for the contusive SCI model. At 14 days, the contusive SCI had increased the TH behavior 54.1% (45.9% of laminectomy. By 21 days, this TH had not improved much (54.7% of laminectomy), but the addition of hNT2.19 grafts had improved TH recovery to 81.8% of laminectomy alone. At 56 days, the SCI alone was only 50.7% of laminectomy, compared to 89.4% fo9r the addition of hNT2.19 grafts the contusive SCI paradigm. Even though the cell lines are different and the SCI models used are categorically different, the effect on TA and TH behaviors with the hNT2.17 and hNT2.19 cell grafts are quite similar. 

#### 3.5.4. Percent Comparison of Open-Field Motor Behaviors after Severe Contusive SCI and Following Either Intraspinal Or Intrathecal Transplant of hNT2.19 Cells

**Figure 9:**
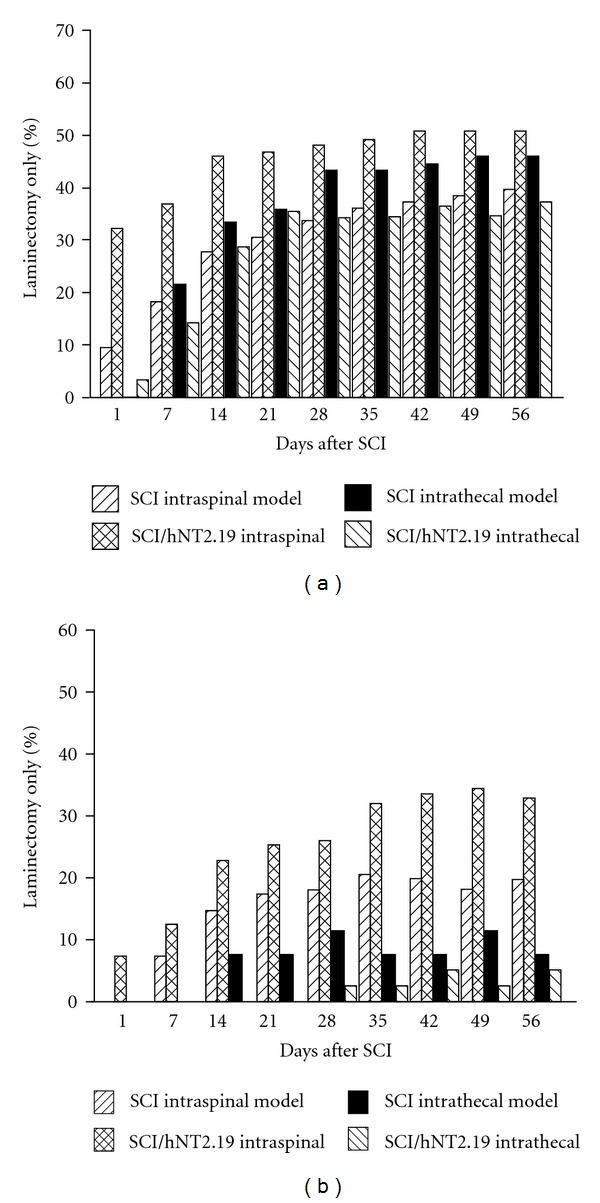
Percent comparison of open-field motor behaviors after severe contusive SCI and following either intraspinal or intrathecal transplant of hNT2.19 cells in vivo: location of graft of hNT2.19. Rats were injured with a weight-drop device (NYU impactor, 25 mm; severe contusive SCI) in a rat model of SCI and chronic motor dysfunction. Some rats received only a laminectomy, and data are a percent of laminectomy control data (for each independent transplant location experiment) for open-field BBB scores (a) and BBB subscores (b) after contusive SCI only and SCI + hNT2.19 cell grafts, where grafts are placed either intraspinally or in the lumbar subarachnoid space (intrathecally). Gross open-field motor behavioral results (a) (BBB) showing gradual recovery of motor scores beginning at 1 week after SCI, with additional partial and persistent recovery with the addition of intraspinal hNT2.19 grafts. There is no improvement over SCI alone when the grafts are placed intrathecally. Data represent the mean value (*n* = 6 animals in each group) at each time point before and for 56 days after SCI. The BBB subscore (b) demonstrated a significant improvement in the subscore, beginning at 2 weeks after SCI, with the addition of the intraspinal, but not the intrathecally-placed hNT2.19 cells. Data represent the mean value (*n* = 6 animals in each group) at each time point before and for 56 days after SCI. Only intraspinally placed hNT2.19 cells improved BBB and BBB subscores.

The grafts of the serotonergic hNT2.19 cells are able to modestly promote the recovery of motor function after severe contusive SCI [46], but it's informative to compare these motor effects when the hNT2.19 grafts are placed at two different locations in the same contusive SCI model. Both the open-field motor behaviors (Figure 9(a)) and fine-motor behaviors (Figure 9(b)) are compared at each week after SCI, and reported as a percent of laminectomy control, when the same dose of hNT2.19 (10^6^ cells/injection) is provided, at either a lumbar subarachnoid or intraspinal site (immediately above/below the lesion site). The open-field BBB score (a) was used to study the functional recovery stages following the injury, by categorizing the rat hindlimb movements, trunk position and stability, coordination, stepping, and paw placement and tail position. The fine-motor BBB subscore (b) described frequency of clearance of toes while moving the hindlimbs and is a measure of improvement which accompanies sufficient improvement in BBB scores. At seven days after the SCI, the SCI had induced 18.3% of laminectomy BBB scores (a) in the intraspinal model. By 14 days, the SCI group had only 27.8% of the laminectomy BBB scores in the intraspinal model. At 56 days, the SCI group had recovered only 39.7% of the laminectomy BBB scores in the intraspinal model. When the hNT2.19 cells are placed intraspinally, recovery reached 46.1% of laminectomy BBB scores (7 days after transplant). By 21 days, recovery improved to 46.8%; by 56 days, BBB scores had improved to 50.81% of laminectomy BBB scores, in the presence of intraspinal hNT2.19 grafts. Results were very different in the intrathecal model for BBB scores (a). At 7 days after SCI, BBB scores were 21.7% of laminectomy for the SCI group; at 21 days, the BBB scores were 36% of laminectomy scores for the SCI group and 35.5% for the SCI/hNT2.19 graft group (7 days after transplant). At 56 days, BBB scores were 46.1% of laminectomy scores for the SCI group and 37.3% for the SCI/hNT2.17 intrathecal transplant group. There was no recovery in BBB scores when the hNT2.19 grafts were placed intrathecally, compared to the modest recovery seen when grafts are placed intraspinally. Similar results are seen with an examination of the data from the BBB subscores (b), and the results normalized in the intraspinal and intrathecal models to percent of laminectomy control. At 7 days after SCI, the SCI had induced 7.4% of the laminectomy BBB subscores in the intraspinal model. By 14 days, the SCI group had induced only 14.7% of the laminectomy BBB subscores, while the addition of intraspinal hNT2.19 grafts had improved the BBB subscores to 22.8% (7 days after graft). By 56 days, when SCI had recovered only 19.7% of the laminectomy BBB subscores, the addition of intraspinal hNT2.19 grafts had improved the BBB subscores to 32.9% of laminectomy controls. When hNT2.19 are placed intrathecally, they never improve BBB subscores above that seen for SCI alone (even lower the subscore, compared to SCI). Overall, only intraspinal hNT2.19 grafts are able to significantly, if modestly, improve BBB and BBB subscores, compared to intrathecal placement of hNT2.19 grafts in the same severe contusive SCI model.

## 4. Discussion

### 4.1. NT2 Cells for Transplant

The teratocarcinoma human NT2 (NT2/D1; hNT2) parental cell line was the source of the hNT2.17 and hNT2.19 cell lines and is derived from the embryonic carcinoma (EC) cell type, after differentiation in response to retinoic acid (RA). A derivative of the original polyclonal TERA-2 EC cell line, hNT2 are cells with phenotypic properties of neurons after differentiation and this resultant, exclusively neuronal, phenotype with RA treatment has remained a hallmark of this human cell line. The RA-differentiated neurons, often called NT2-N cells, are similar to developing human spinal cord or CNS neurons, reminiscent of terminally differentiated postmitotic neurons. Such neurons express typical neuronal markers [82], with a stable polarized phenotype [83] of central, not peripheral nervous system neurons. Spinal implantation of differentiated hNT2 cells in rats results in grafted neurons with 1-2% survival (with brief immunosuppression) at three months after transplantation. These surviving neurons display a robust axo-dendritic sprouting and expression of markers typical of mature neurons and grafted neurons with a mostly GABA phenotype [64]. More recently, the same hNT2 cell grafts uniformly showed robust cell survival and progressive neuronal maturation, with most transplants demonstrating a GABA phenotype, after injection into the lumbar spinal cord segments of naive immunosuppressed minipigs [84]. Both the hNT2.17 and hNT2.19 cells, derived from this hNT2 lineage, have been sufficiently characterized in vitro and in vivo to determine their exclusive neuronal and neurotransmitter phenotype [43, 47, 61].

#### 4.1.1. Phenotype of hNT2-Derived Cell Lines

The fact that the parental hNT2 cell line contains multiple, distinct cell types, and hence exists more as a cell population rather than as a pure cell line, serves as an advantage when these cells are intended to be subcloned into multiple and distinct cell lines. A few characterization studies [85, 86] describe a variety of phenotypes possible, including cholinergic, GABAergic, catecholaminergic, serotonergic, and peptidergic, expressed by hNT2N neurons after 2B4 weeks of differentiation in vitro. The most common phenotype observed in the parental hNT2 neurons is the GABAergic type (about 62% in [85]; about 15% in [86]). The other common phenotypic markers seen in parental hNT2 neurons were glutamatergic or dopaminergic, but in our hands these neurotransmitters were not seen in either the subcloned hNT2.17 or hNT2.19 cells. Another common hNT2 phenotype, depending on the cell preparation method, was for 5HT-expressing hNT2 cells, ranging from about 2% [86] to 30% [85]. Further increasing the proportion of 5HT producing neurons seems to require particular differentiation protocols involving the timed application of various growth factors [87], methods not used in the current subcloning of the hNT2.19 cell line. Our differentiation method is similar to that of the Guillemain study [85], which provides about 30% 5HTcontaining neurons. This explains the relative ease of finding both a GABA or 5HT-subclone, such as the hNT2.17 and hNT2.19 cell lines, since our methods for subcloning depended on examining single cells which formed colonies (and established the subsequent cell lines). Using the rapid aggregation method for differentiation [82], and determination of good cell survival and doubling times in culture, as well as anti-neurotransmitter antibody markers for the choice of candidates for useful cell lines, meant finally screening about 100–150 cell lines, to derive a few hNT2-lines, and finally the hNT2.17 and hNT2.19 cell lines. A similar approach could only be hurried with the use of automated mechanical cell-picking devices for subcloning, rather than the laborious art of using cloning rings and individual cell line analysis, as was done here. 

There is much interest in directing the eventual and terminal phenotype of stem cells [88], and, interestingly, the parental hNT2 cell line (see: patent application US 2008/0160614A1, Stable Differentiation of Adult Stem Cells, Saporta et al.) without the use of growth factors or retinoic acid, since the growth factors and/or retinoic acid can be difficult to completely remove during commercial production for clinical use. These efforts are based on the observation that particular phenotypes expressed in differentiated hNT2 cells (usually neurons) do not appear to be stable (beyond 30+ days in culture). In our hands, the GABA phenotype (of hNT2.17) and 5HT phenotype (of hNT2.19) remain 100% after 30+ days in vitro and >6 weeks after grafting [43] into the lumbar subarachnoid space, but with further studies, that phenotypic instability observed by others may indeed prove true. Alternative methods for generating a specific, and useful, phenotype, at least in immortalized cell lines of murine origin, often involve both immortalization with an oncogene [89], such the temperature-sensitive mutant of SV40 large T-antigen (ts-Tag) [90], as well as the possible addition of a rate-limiting neurotransmitter enzyme gene, such as GAD or TH, or addition of (or in vitro exposure to) a neurotrophin gene such as BDNF, to drive terminal differentiation. Such studies and methods have occupied our interests for years [16, 28–30, 91–98], in fact leading to the conclusions that GABA and 5HT were the most effective agents for anti-nociception in cell therapy (with studies of bio-engineered rat cell lines), but neither cell-transfection technology nor non-human cells will likely ever be useful (or meet FDA approval; see: [99]) for safe human cell therapy. Perhaps only the simplest manipulations, stem cell sources or otherwise, for production of cell sources for cell therapy will meet current approval. The parental hNT2 cell line has of course been approved for clinical trials [40], and it might be expected that these hNT2-derived and subcloned cell lines could also meet FDA approval. But bio-engineering cells, tailoring cells to meet specific phenotype requirements, with current gene transfection vectors, await thorough vetting for safety, similar to that seen in the development of new gene therapy approaches [100].

Additionally, the colocalization (and release) of other neurotransmitters which might be relevant for anti-nociception, that is, glycine in hNT2.17 cells [43, 61], or a specific synaptic amino-acid transporter, such as vesicular inhibitory amino acid transporter (VIAAT), also found in hNT2.17 cells [43], suggests that these hNT2-derived cell lines are mature, functional, human neurons, and likely remain so after grafting in a murine or human host, able to secrete or release neuroactive agents into the neural environment, and they also will likely integrate into the neural matrix, when conditions are appropriate, as has been seen for the hNT2 parental cells [65, 101]. Our single study of intraspinal transplant of the differentiated serotonergic hNT2.19 cells [46] demonstrated a modest effect on recovery-of-motor function after SCI with grafts, without easily revealing surviving grafted cells (in the nude rat). Further studies will be needed to understand how well these two cell lines integrate into white- and grey-matter in the PNS or CNS, but they clearly maintain their specific neurotransmitter phenotype when placed in a subarachnoid location, near the lumbar cord.

#### 4.1.2. Tumorgenicity Considerations

Part of the concern for safety in these cell lines for clinical use [37] is the issue of expression of a tumorigenic phenotype in the human host. Postmortem examination of a single patient following parental hNT2 graft for stroke showed that no tumor was identified anywhere in the brain, and a monoclonal antibody to Ki-67, a protein expressed in cycling cells, immunolabeled 1% of cells, consistent with the absence of a neoplasm [65]. These hNT2 cells which require differentiation before grafting to eliminate the possibility of tumor formation, have been well-studied in animal models [38, 39]. Other studies provide some understanding of the central role of retinoic acid regulation of differentiation [35, 102] and suppression of tumor genes, such as FGF-4 [103] and TGF-*α* [103, 104], in RA-sensitive cell lines, such as the hNT2. Downregulation of FGF-4 and TGF-*α*, as well as expression of cytoskeletal proteins [83], serves as markers of differentiation with lack of tumorogenicity, and FGF-4 and TGF-*α* are expressed in both the proliferating, but not differentiated, parental hNT2 [105] and our hNT2-derived [43, 47] cell lines. With RA-exposure these cell lines terminally differentiate, express a number of markers of mature neurons [43, 47, 61, 83], with FGF-4 and TGF-*α* effectively inhibited in differentiated cells. In addition, immunohistochemistry for the BrDU marker in proliferating versus differentiated cells, shows similar results, for example, the hNT2.17 and hNT2.19 cells used for grafting do not take up BrDU, and the overall conclusion is that these cell lines are not tumorigenic when used after RA-exposure and differentiation, when placed within or outside of the spinal cord. Over more than 10 years of using these two cell lines as graft sources in naïve and immunosuppressed rats, with and without chemical- or mechanically-induced injuries, no rats have ever developed tumors or further mechanical or sensory impairment, even in animals followed out to 3 months after cell placement. The hNT2-derived cells were always well-differentiated (for at least 2 weeks following 2 weeks of retinoic-acid treatment), exclusively neuronal in phenotype, with no reversion to a tumorigenic (genetic) phenotype. Histopathological examination [10] of the subarachnoid and spinal cord transplant site in a quisqualic (QUIS) lesion SCI paradigm concluded that hNT2.17 (grafted) cells were identified on H+E staining as small round basophilic cells without significant dendritic or axonal processes. The H+E staining showed no difference between the cytoarchitecture of QUIS and QUIS plus transplant and showed no cord damage in the naive plus transplant cord group, compared to naive. Myelin staining showed significant demyelination in all animals undergoing a QUIS injury that did not improve with hNT2.17 transplants. No demyelination was seen in conjunction with cell grafts, implying their safety as a graft source Such long-term data will likely be required in mouse tumor studies under GLP conditions for eventual clinical use.

### 4.2. Survival of Grafted Cells

Graft survival and surviving cell numbers have been examined in a series of studies of the hNT2.17 cell line grafts in the excitotoxic QUIS model of SCI pain [43, 61]. With their identity as a model of human neurons, it is not surprising that the hNT2 cell line has been used to examine a variety of genetic elements that can influence cell survival, for example, RA-induced activation of the p53 gene [106], or expression of the neurotrophin BDNF [107] in hNT2 cells, suggesting that these factors likely drive early neuron survival in normal brain development. Using the transplantation of hNT2 cells into the CNS of immunodeficient mice as an in vivo model system for studies of the formation and remodeling of the developing central nervous system [108], survival, and integration of undifferentiated grafted cells are seen to depend on graft site, suggesting that areas of the brain, such as the caudoputamen, contain environmental cues that lead to the progressive differentiation of large numbers of NT2 cells into postmitotic, immature, neuron-like cells, whereas transplant of undifferentiated cells into the subarachnoid space induces both tumors and massive cell death. When hNT2 cells are predifferentiated with RA, the bcl-2 gene expression is upregulated and after transplant into the rat striatum, 85% of implanted neurons expressed bcl-2, with 12% of the hNT2 neurons surviving the transplantation at 1 month. Collectively, these data suggest that only predifferentiated hNT2 cells can be used for transplant and RA induction is required for differentiation before grafting. In our experience, both pre-differentiation and an optimal course of CsA immunosuppression around the time of transplant are required for good graft survival in the subarachnoid space [43]. Since transplant into a nonhuman host such as the rat, these xenografts, require at least 1-2 weeks of immunosuppression. The most interesting data comes from examination of graft sites from either intraspinal transplant of hNT2.19 cells in contusive SCI [46], or cervical subdural grafts of hNT2.17 cells in QUIS SCI [61], where motor and sensory recovery does not require long-term hNT2.17 graft survival, since transplanted cells could not be identified at the 6–8 week experiment's end. But even optimal survival of these xenografts in a subarachnoid location was about 1% after 8 weeks with SCI [43], suggesting that at least some course of immunosuppression might be required in humans, or repeated, additional i.t. injection of antinociceptive hNT2-derived cells would be necessary if effectiveness is reduced over time in humans, as has been observed with i.t. injection of anti-nociceptive chromaffin cells for terminal cancer pain in Phase II trials [109].

### 4.3. Requirement for Immunosuppression

Requirements for immunosuppression of i.t. cell grafts suggest complex issues [110], even with toxicity, such as nephrotoxicity, hypertension, hypertrichosis, infection, hyperkalemia, and neuropathy, associated with CsA use and cell transplant [111]. There is a contention of the CNS being a “privileged transplant site,” but the issue is controversial, even with the use of allogenic stem cells transplants into the nervous system [112]. But some trends are seen with the hNT2.17 grafts in the QUIS model of pain: a minimal or optimal course of immunosuppression with CsA, about 1 to 2 weeks after transplants, is required; this minimal CsA course ensures optimal efficacy in reversal of the behavioral hypersensitivity associated with SCI-pain; less than minimal immunosuppression (1 day) only provides minimal efficacy; longer than the optimal time course of CsA does not improve efficacy significantly. The differences in recovery of TA versus TH when less than 2 weeks of immunosuppression are provided is also interesting to note. A mixed CsA effect occurs after 1 week of treatment and seems to preserve the antiallodynic effects of the transplants but not the antihyperalgesic effects. Certainly the grafts do not survive as well as when 2 weeks of CsA is provided for immunosuppression. We examined immunostained sections at the end of these experiments (data not shown), and although reliable quantification of grafts is almost impossible, there were clearly fewer surviving grafts with less than 2 weeks of CsA. A “critical” number of functioning grafted cells could influence or permanently affect TA versus TH. Also, TA is likely modulated differently than TH; these differences are also seen in the timing studies. We have seen similar differences in previous studies of the effects of timing with rat cell therapy and the CCI peripheral model of neuropathic pain [113].

### 4.4. Timing of Graft Placement

The issue of altered efficacy with changes in the time of placement of cell grafts was first examined with the use of GABA-secreting cell grafts in the CCI-model of peripheral nerve injury pain and the GAD67-rat cell line [113], where lumbar subarachnoid transplant of these cells only permanently and completely attenuated TA and TH behaviors induced by the unilateral nerve injury when an early time point (2 weeks after CCI) was used for cell injection, even though many surviving grafted cell were found, no matter when cells were grafted. A similar result is seen with the use of hNT2.17 lumbar grafts in the QUIS model of SCI pain [61]. In both studies, even with the great differences in the rat pain models and the characteristics of these GABA-secreting cell lines (rat and human derivations; bioengineered versus “natural” GABA phenotypes), the profile of effects is remarkably similar. Early transplant times (2 weeks after nerve or spinal cord injury) provide complete and permanent attenuation of behavioral hypersensitivity. Later transplant times (6 weeks after nerve or spinal cord injury), when both TA and TH behaviors are well established has, at best, a temporary and/or partial effect. Although a percentage of patients report neuropathic pain immediately with injury, onset time is often 3–6 months later with both peripheral and CNS injuries. Another common observation is that while acute neuropathic pain more often responds to treatment, chronic neuropathic pain can be resistant to a variety of treatments [114]. Where pharmacologic pain treatment is effective, onset of relief can be immediate [115], but these same medications, where effective, are best in chronic pain and must be individualized to specific pain scenarios, with the appearance of side-effects at higher doses [77]. Our data suggest that the earlier the initial GABA hNT2.17 grafting the better the outcome, although even a graft provided 6 weeks after SCI in the rat provided >70% behavioral efficacy. Understanding the mechanisms in both the initiation and maintenance of pain behaviors in any type of pain, as well as for it=s relief, are the keys to understanding any eventual use of cell therapy in humans.

### 4.5. Graft Location in Recovery-of-Sensory Function

For decades, development of the idea of cell therapy for pain has envisioned and tested the lumbar subarachnoid location for anti-nociceptive transplants [11, 116]. Whether primary cell sources, human [117] or xenografts [118], stem cells, immortalized cell lines [30, 119, 120], or encapsulated cell or tissues for immunoprotection [121], cell transplants have usually been examined for their effects on lumbar dorsal horn sensory systems in models of pain. But several epidemiological studies suggest that most or many SCIs are at a cervical spinal level [122], although chronic pain is a common report following thoracic SCIs [123]. Most studies report pain in distal limbs, even following clinically complete injury. But neuropathic pain, associated with SCI [124] or peripheral injuries [125] in upper limbs is also a common complaint, such that testing graft location, cervical as well as lumbar, in cell therapy studies in a SCI pain model is a reasonable preclinical approach. Here a cervical graft of GABA hNT2.17 cells completely reverses behavioral sensitivity measured in the hindlimbs equally as well as a lumbar graft. Although these differing graft sites were examined some 6 weeks after transplant with no surviving cervical grafts visible, such cervical grafts were equally efficacious for the behavioral hypersensitivity from a low thoracic SCI. Although the lumbar i.t. space is larger than the cervical space, it has relatively stagnant CSF flow to compensate for intraspinal/intracranial pressure changes. CSF flow is greatest in the cervical space, perhaps a result of the “bottleneck” at the entrance to the brain. However long the grafts might survive on the cervical pia (to produce identical behavioral effects as lumbar placement), by the end of the experiment, they will have likely eroded into the CSF flow. Similar results are seen on both forelimb and hindlimb behavioral hypersensitivity, when cell grafts are placed in a lumbar subarachnoid location [118], and it is logical to expect that upper-limb neuropathic pain could be attenuated by lumbar grafts of hNT2-derived cells.

### 4.6. Relief of Dysesthetic Pain Behaviors in Excitotoxic SCI

One common feature in the excitotoxic SCI pain model is the “excessive grooming” or autophagia the spinal injection of quisqualic acid produces in the animals' hindlimbs [126], where cell therapy [55] or antiimmune modulation [127] is seen to reverse the behaviors and reduce or eliminate the lesions. When excessively grooming rats that had been transplanted with either viable or nonviable hNT2.17 cells and exposed to different immunosuppression regimens were examined for development, resolution, worsening, or no change of excessive grooming, a trend toward improvement was associated with viable grafts and at least 1 week of accompanying CsA immunosuppression. When transplant was delayed to 6 weeks, no improvement in excessive grooming was seen. Since previous studies [128] have suggested that the amount of tissue damage at the epicenter of injury is critical to the onset of spontaneous overgrooming behaviors, early transplant of hNT2.17, with rapid resolution of TA and TH behaviors, may also be neuroprotective, by some currently unknown means, and the critical threshold of tissue damage required for the onset of over-grooming behavior may never have been reached. Such a hypothesis suggests that spinal lesion analysis in SCI with these human cell grafts would be a fruitful line of research to examine, especially with concomitant measure of spinal neurotrophin levels [129], as has been seen with other treatment approaches in SCI.

### 4.7. Use of Grafts in the Peripheral Nerve Injury and DPN Pain Models

Peripheral nerve injury can produce significant neuropathic pain [130], and although diabetic peripheral neuropathy (DPN) is a common medical condition that often induces neuropathic pain, mechanisms are poorly understood [131] in these injuries and disease states. With disinhibition, or loss of inhibitory control proposed as part a possible central mechanism for peripheral neuropathic pain [24, 132], transplant of GABA-secreting hNT2.17 cells was tested in the CCI and DPN models of pain. Creation of rat cell lines, bioengineered to synthesize and secrete GABA or 5HT, have been tested previously in the peripheral nerve injury CCI model [29, 30, 113], and use of the human GABA hNT2.17 grafts to relieve neuropathic pain in the DPN pain model has been reported preliminarily [133]. Here, transplant of the human hNT2.17 cells two weeks after unilateral CCI, potently and permanently attenuated both TA and TH behaviors. Interestingly, when these data are compared with the grafts of predifferentiated parental hNT2 cells, attenuation is partial (in the case of tactile allodynia) or less immediate and robust (in the case of thermal hyperalgesia), compared to grafts of the subcloned hNT2.17 cells. It is likely, considering that the GABAergic phenotype in the hNT2 represents only about 50% of the total and if the sensory recovery is based on the presence of GABA-secretion from transplants, that the improved attenuation of the hNT2.17 grafts may be due to the 100% GABA expression in the subcloned hNT2.17 cells [43] after transplant into the subarachnoid space. When these data are compared to a recently published report, from an independent investigator, of grafts of the hNT2.17 in the CCI model of pain [45], with some differences in the method of TA analysis, the profile of sensory recover-of-function are remarkably similar. In this study, the two week after nerve (CCI) injury was the same for transplant placement, as was the cell injection dose (10^6^ cells/injection). In this latest study, the presence and GABA phenotype of the grafted hNT2.17 was easily detectible at the end of the experiment (4 weeks after transplant), and nonviable grafts of hNT2.17 had no effect of TA and TH behaviors, as was seen in our own studies, either with nonviable hNT2 or hNT2.17 cells. This independent confirmation of attenuation of TA and TH behaviors argues for the use of a human GABA cell line, such as the hNT2.17 cell line, for the relief of peripheral neuropathic pain, at least that modeled by the CCI animal model.

Examination of grafts of the GABA hNT2.17 cells in a model of DPN pain offers another example of the potential usefulness of these cell lines. This animal model utilizes a single i.v. injection of streptozotocin (STZ), which makes the animal profoundly and permanently hyperglycemic. This in turn leads to measurable neuropathy and behavioral hypersensitivity to tactile and thermal stimuli. These outcomes parallel those observed in human diabetic peripheral neuropathies [134]. Exact mechanisms for neuropathic pain in DPN are unknown, but aberrant spinal or supraspinal modulation of sensory processing may be involved in generating the tactile allodynia and thermal hyperalgesia in the STZ model. Studies have supported a role for spinally mediated hyeralgesia in diabetic rats that may reflect either a response to diminished peripheral input or a consequence of hyperglycemia on local or descending modulatory systems [135]. The strategy for transplant of islet cells, whether autologous, allogenic, or xenografts, in the STZ model, that normalizes blood glucose levels, also reduces TA and TH [136] and has been tested in human trials [137], with mixed results. The association of reduced blood glucose and prevention/reduction of DPN and pain is a long-standing concept in the treatment of diabetes [138]. The multifactorial causes of DPN pain, including increased insulin resistance, oxidative stress, increased sorbitol, decreased nitric oxide, and increased homocysteine have been identified as the primary factors involved [23], and to date a few pharmacologic agents have improved the quality-of-life and provided pain relief, but have side-effects at higher doses [139]. Here, we show in the STZ model of DPN pain, that not only do the transplant of GABA hNT2.17 survive and express their GABA phenotype in the subarachnoid space for 6 weeks (with limited CsA immunosuppression), but they attenuate both TA and TH behavior, induced by STZ injections. However, such grafts do not ameliorate the STZ-induced hyperglycemia, suggesting that pain reduction and blood glucose control are likely controlled by separate mechanisms. There does seem to be a trend towards recovery of body weight (data not shown) associated with recovery-of-sensory-function, but it may only suggest that the animals' appetite stimulation is related to improved sensory function. However, with no data clearly demonstrating that preventative or potentially curative measures can reduce the incidence of neuropathic pain in diabetic patients [140], this use of a human neuronal cell line might be an alternative or adjunctive approach to treat neuropathic pain, especially in type 2 diabetes where insulin may not be used.

### 4.8. Use of Grafts in SCI Models of Pain and Motor Dysfunction

The transplant of the GABA hNT2.17 cell line in the excitotoxic QUIS SCI model of pain has been reported previously [43, 44, 141, 142], but we have not reported on a direct comparison of hNT2.17 grafts with those of the 5HT hNT2.19 cell line in the same model. Although, even performed in the same model, with the lumbar injection dose the same (10^6^ cells/injection), it might not be expected that such dissimilar cell phenotypes would result in replicate data. Actually, there is no significant difference in the attenuation of TA and TH behaviors induced by these two cell lines. Each produces potent and permanent attenuation in the QUIS model of SCI pain. This may suggest that recovery-of-sensory-function may be partially amenable to both GABA and 5HT anti-nociception.

When both the QUIS and severe contusion models of SCI pain, certainly very different in etiology, are used to compare grafts of hNT2.17 (in the QUIS model) and hNT2.19 (in the severe contusion SCI), the previously published data [47, 61] normalized as percent of laminectomy control reveals similar effects (percent recovery-of-sensory function). Each cell line, in each SCI model, attenuates TA and TH behaviors equally and with the same temporal profile, compared to SCI alone. In each case, surviving grafts keep their specific neurotransmitter phenotype at the end of the experiment (as seen in graft site examination). Again, this suggests both a GABAergic and serotonergic mechanism for recovery-of-sensory-function in these unique models of SCI pain, if indeed the mechanism-of-action for each cell line is related to their neurotransmitter phenotype.

We took a similar approach here to an examination of motor recovery of previously published data [46, 47], by normalizing the percent of open-field and fine motor-recovery with the grafts of the same cell line, the 5HT hNT2.19 cells, in the severe contusive SCI model of pain and motor dysfunction, when the transplants are placed in either an intraspinal or an intrathecal graft location. Only in the case of intraspinal hNT2.19 grafts was cell transplant able to modestly improve postgraft motor dysfunction. Such results suggest that the lack of diffusion of 5HT able to penetrate the spinal cord with lumbar i.t., hNT2.19-based, secretion of 5HT [143], and the improved response of motor systems to ventral spinal 5HT applications in SCI [144, 145], the location of any cell therapy should consider the accessibility of the endogenous cellular systems to be affected. We have seen similar location-specific effects when a rat cell line, bio-engineered to secrete 5HT, was used in the motor/sensory hemisection SCI model [146]. Such rat cell line 5HT grafts improved the endogenous motor and sensory 5HT systems in spinal hemisection [147, 148], regulating the intrinsic spinal 5HT systems, increasing the BDNF spinal content, and reducing bilateral hyperexcitability in the dorsal horn, and it might be expected that grafts of human 5HT hNT2.19 work by the same or similar mechanisms in contusive SCI. 

### 4.9. Mechanisms

#### 4.9.1. The GABA Hypothesis for Chronic Pain and Relief by Cell Therapy

In the spinal cord, GABA is concentrated in interneurons of the superficial dorsal horn, laminae I–III, the main site of termination of A*δ*- and C-fiber afferents, where GABA-containing interneurons presumably function as inhibitory [149] to sensory afferents. In the spinal cord, GABA acts at either the ligand-gated chloride channel GABA-A receptor or the G-protein-linked GABA-B receptor. Both receptors are present pre- and postsynaptically on A*δ*- and C-fiber afferents [150], each with specific agonists and antagonists [151] acting preferentially. Many clinically relevant drugs directed against the GABA-A receptor, such as the benzodiazepines, interact at additional allosteric-binding sites, referred to as the GABA-A/benzodiazepine receptor complex [152].

A number of animal models have been used to study SCI and produce chronic pain, including photochemically induced ischemia; hemisection of the spinal cord [153]; excitotoxic lesions using intraspinal injections of excitatory amino acid agonists [78, 154]; spinal contusion injury. All these models, even with significant differences in their mechanisms of onset and duration of pain behaviors, have at least one common explanation for the changes in intraspinal biochemistry to account for the chronic pain that they produce, namely, the loss of inhibitory tone, or loss of modulation by the GABA-releasing interneurons in the cord after injury [49, 155–157] consistent with the development of neuropathic pain following SCI. This is further supported by the loss of these neurons following injury [49, 157] and the fact that the behavioral changes, that is, allodynia, produced by injury can be induced by the intrathecal administration of GABA antagonists [156, 158]. On the other hand, i.t. administration in the lumbar spinal cord of GABA-A or GABA-B receptor agonists attenuated SCI-induced mechanical allodynia in both hindpaws in spinal hemisection [159]. Efforts to restore GABA levels by pharmacological intervention has proven effective but difficult to manage [160], whereas spinal GABAergic systems that can be restored by cell therapy with i.t. injection of cells, such as bioengineered cells [55], adrenal chromaffin tissue [161], and now human neuronal GABA cell lines such as hNT2.17, seems to be a viable and practical approach.

#### 4.9.2. Nerve Injury and GABA for Pain

Neuropathic pain following peripheral nerve injury, such as the Bennett and Xie sciatic CCI model [53], also appears to be subject to GABAergic control. Intrathecal administration of the GABA-A receptor antagonist, bicuculline, caused a dose-dependent increase in the magnitude of hyperalgesia when given within a few days of CCI [162]. In a similar nerve injury model that includes the ligation of the L5/L6 nerve roots [163], intrathecal injection of both the GABA-B agonist baclofen and the GABA-A agonist muscimol resulted in a dose-dependent antagonism of the induced tactile allodynia two weeks after injury [132], suggesting the both GABA receptor types modulate spinal systems activated by low threshold mechanoreceptors which mediate tactile allodynia following peripheral nerve injury.

The behavioral changes, that is, allodynia, produced by nerve injury can be induced by the intrathecal administration of GABA itself [74] or GABA receptor antagonists [164], and is reversible by specific receptor agonists [165]. In animals with acute mechanical allodynia, the hypersensitivity of spinal wide-dynamic range neurons to mechanical stimulation was reversed by the GABA-B receptor agonist baclofen, but not by the GABA-A agonist muscimol after ischemic SCI. However, baclofen failed to relieve the chronic mechanical allodynia that was present several weeks after this ischemic injury [166].

But mechanisms for behavioral hypersensitivity in the CCI and other models of neuropathic pain are poorly understood. CCI (and other nerve ligation/injury procedures) promote a functional loss of GABAergic transmission in the superficial dorsal horn via reduction of presynaptic GABA release after CCI. Partial nerve injury also decreases dorsal horn levels of the GABA synthesizing enzyme glutamic acid decarboxylase (GAD) 65 kDa ipsilateral to the injury and induces neuronal apoptosis, detected by terminal deoxynucleotidyl transferase-mediated biotinylated UTP nick end labeling staining in identified neurons [167]. At the same time, tactile allodynia [168] and thermal hyperalgesia [169] can occur in the CCI model without selective loss of GABA or GABA(A) receptors, the expression or function of GABA(B) receptors in spinal cord and dorsal root ganglia [170], from synapses in laminae I-II of the ipsilateral spinal dorsal horn.

When cellular therapy using subarachnoid transplants of adrenal chromaffin cells [161] or raphe cell lines that secrete GABA or serotonin are used, rather than pharmacologic interventions, in models of chronic pain after SCI [95] and sciatic nerve CCI [161, 171], grafts that reverse chronic pain after nerve injury are able to restore the loss of the endogenous spinal GABAergic system in the dorsal horn pain processing centers within one to two weeks after grafting [171]. As well, partial nerve injury causes a slow upregulation of glutamate decarboxylase-67 (GAD67) synthetic enzyme expression in these same endogenous cells related to a decrease of GABA synthesis. This upregulation of GAD67 expression is also reversed by the cell grafts [171]. The endogenous GABA interneurons that modulate neuronal hyperexcitability in the pain pathways contain very high levels of GAD67, rather than the GAD65 synthetic enzymes for GABA synthesis [172]. Of these two enzymes responsible for GABA synthesis, GAD67 is exquisitely sensitive to GABA levels [173], although it is not clear at what point in the pathway, either the protein or message ribonucleic acid (mRNA) for the GAD67 enzyme, that endogenous GABA levels, or other cellular pathways related to pain, exhibits control of the enzyme. The measured changes in GAD activity with manipulation of endogenous GABA content are attributable to changes in GAD expression [174], rather than loss of enzyme activity, but whether similar mechanisms function in chronic pain is not clear at this time. When the endogenous dorsal horn GABA interneurons are examined after partial nerve injury and neuropathic pain, the greatest loss of GABA-immunoreactive (ir) neurons (and presumably synthesis of GABA) is at two weeks after nerve injury [161]. Such loss of GABA-ir does not ever fully recover on the ipsilateral side [161, 171], even though the ipsilateral paw often demonstrates much less or no tactile and thermal hypersensitivity at eight to nine weeks after nerve injury [53]. We have observed a similar loss of GABA-ir neurons 2 weeks after injection of QUIS, especially ipsilaterally (unpublished observations). This permanent loss of GABA-ir suggests an ongoing remodeling, and possibly permanent disappearance, of elements of the GABA system that modulate afferent/efferent information into and out of the dorsal horn, as has been seen by others in SCI [157]. There is not yet clear evidence for a downregulation, or decrease in numbers, of GABA receptors after nerve injury, although there is a recent suggestion for the uncoupling or dysfunction of GABA-B receptors following unilateral hindlimb axotomy [175]. Since the expression of GAD67 enzyme, likely responsible for long-term alterations in GABA synthesis, is apparently altered, and probably upregulated, after nerve injury and chronic pain [171], the ability of cell therapy to reverse behavioral hypersensitivity after injury when GABA synthesis is lowest in the lumbar dorsal horn is hypothesized to be related to this change in GAD67 expression. Such studies suggest that early successful intervention for pain may involve restoration of that endogenous GABAergic system [30] with cell therapy. Interestingly, a single bolus injection of intrathecal GABA [74], if injected within a few weeks after nerve injury, completely and permanently reverses behavioral hypersensitivity after partial nerve injury. Recently [45], it has been shown that intrathecal grafts of the GABA hNT2.17 cell line in the CCI model of pain caused a strong induction of GAD67 mRNA with one week after graft, which was followed by a recovery of GAD67 and GABA-ir. This effect paralleled a reduction of hindpaw hypersensitivity and thermal hyperalgesia induced by CCI. In the same study, and in concert with our studies, the decrease in GABA expression in the spinal dorsal horn of injured animals is concomitant with a decline of its synthetic enzyme GAD67 immunoreactivity and mRNA but not the GAD65 enzyme. Graft of GABA hNT2.17 cells induces a GABAergic mechanism in the partial nerve injury model of pain and may induce a similar mechanism in other peripheral and central pain models of pain and injury. As shown here, grafts of these cells potently and completely reverse TA and TH behaviors in diabetic neuropathy pain, and excitotoxic SCI pain. Since 5HT cells of rat origin reverse neuropathic pain, at least partially, by a GABAergic mechanism in nerve injury [171], it is possible that grafts of 5HT hNT2.19, which reverse pain behaviors in SCI models of pain as shown here, may also induce the same or similar GABAergic mechanisms to reverse neuropathic pain in SCI models of pain.

#### 4.9.3. Diabetes and Chronic Pain

Diabetic neuropathy, with neuropathic pain, is a common complication of diabetes. It usually progresses gradually and involves small and large sensory fibers. The symptoms, such as loss of ability to sense pain, loss of temperature sensation, and developing neuropathic pain, follow a “glove and stocking” distribution, beginning in the lower limbs, first affecting the toes, and then progressing upward [176]. Painful symptoms reported by patients with diabetic neuropathy have been frequently documented. Neuropathic pain symptoms are reported in 3–20% of patients with diabetic neuropathy [177, 178]. Features of neuropathic pain such as pain paroxysms, deep aching pain, and hot or burning pain have often been described [179, 180]. In the clinical setting, management focuses on two aspects: disease modifying treatment such as glycemic control and the use of various kinds of analgesics to reduce the intensity of the pain. Although pain intensity may not be sufficient to reflect the outcome of treatment, it is a common outcome measure in clinical research with DPN. Few studies reported treatment efficacy for different qualities of pain such as allodynia and burning pain. Apart from glycemic control, antidepressants and anticonvulsants are commonly used to reduce the intensity of pain in patients with painful diabetic neuropathy. In the clinical setting, despite the use of various analgesics to manage the neuropathic pain of diabetic neuropathy, the problem persists. Such therapies in combination with nonpharmacologic strategies appear to be the future of pain management [181].

Recent results with cell transplants for diabetic neuropathy [182] and its angiogenic effects in a pain-related model of DPN, as well as islet-cell grafts to ameliorate the complications of DPN [136], suggests that cell therapy might be a meaningful therapeutic strategy for the large proportion of DPN patients that also report intractable neuropathic pain. Our own data, presented here, suggest that DPN pain may involve, at least partially, GABA-related disinhibition at the spinal level of the lumbar cord which is able to be reversed by early grafts of hNT2-derived cell lines such as hNT2.17 cells. What is yet to be tested is whether the 5HT hNT2.19 cell line may have the same potential to affect DPN pain, and especially, whether any of these cell therapy approaches would be useful in the chronic forms of DPN pain.

#### 4.9.4. A Role for Serotonin as Antinociceptive in Chronic Pain Following SCI

Supraspinal inhibitory pathways that project to the dorsal horn include those that supply the monamines, serotonin (5HT), norepinephrine, and dopamine [183–185]. Of these, 5HT is the best studied inhibitory neurotransmitter in SCI. Descending serotonergic pathways originate in brainstem raphe nuclei and terminate in the superficial dorsal and ventral horns of the cord [186–188]. Evidence supporting a role for 5HT in nociception [189–191] is based on its anatomical location, the behavioral effects of intrathecal serotonergic drugs [191–193], and inhibition of spinothalamic tract cells involved in pain transmission [194]. Loss of 5HT acutely after SCI caudal to an injury site is a consistent report [195] in a variety of SCI models including deafferentation [196], spinal hemisection [148, 197, 198], and more recently, clip-compression injuries of the cord [199]. This loss of 5HT after SCI has been used as an indicator of injury severity [200]. After hemisection, with injury-induced tactile allodynia and thermal hyperalgesia, animals develop hypersensitivity to lower doses of intrathecal 5HT for antinociception, related to specific 5-HT_1A_ and 5-HT_3_ receptors in the dorsal horn [198]. Grafts of rat cells that release 5HT into the intrathecal space following dorsal hemisection restore spinal 5HT in the dorsal horn [148], increasing 5HT in the CSF, and correct membrane hyperexcitability and phenotype shifts of dorsal horn neurons [96] associated with tactile allodynia and thermal hyperalgesia following SCI [95]. These same 5HT cell grafts are not antinociceptive when placed within the cord, rather than a subarachnoid location, in the same injury paradigm [146], arguing for a focal application of serotonin to/near the dorsal horn (e.g., the subarachnoid space), without further disturbance to the cord, that is required for an antinociceptive strategy with a cell-based approach. Our own data with the use of the 5HT hNT2.19 transplants shown here, and in previous publications [46, 47], demonstrates that intrathecal graft, cell-based approach well in two animal models of SCI pain.

#### 4.9.5. A Role for Serotonin to Enhance Motor Recovery of Function after SCI

Endogenous and exogenously applied serotonin modulates the motor system and stimulates motor recovery after SCI [201], with 5HT agonist application seen to directly depolarize *α* motor neurons [202]. The central pattern generator(s), the local circuitry responsible for rhythmic control of limb movements is modulated by descending serotonergic inputs [203], where the 5HT spinal innervation is eliminated below the level of SCI following injury. Following spinal transection, rhythmic locomotor function and increased responsiveness to reflex testing can restored by transplant of embryonic serotonergic raphe cells [204], presumably by replacement of synaptic connections to motor neurons and release of 5HT in the immediate spinal environment [144]. Our earlier published data, using grafts of rat 5HT cells derived from an immortalized 5HT cell line [95], locomotor function can only be improved after SCI when grafts are placed within the cord; the same grafts are effective for antinociception are effective when placed in the subarachnoid space [146], by attenuating the neuronal hyperexcitability induced by SCI in the dorsal horn [96]. The effects on motor neurons by 5HT-secreting intraspinal grafts is presumably induced by increasing excitability of host neurons through increases in amplitude of monosynaptic reflexes in the central pattern generator circuitry [205], given the excitatory role of 5HT in the ventral horn [202], as opposed to the indirect inhibitory role for 5HT in the dorsal sensory horn system [206]. Agonists for 5HT facilitate, rather than directly generate, stepping, by enabling the spinal cord neural circuitry to process specific patterns of sensory information associated with weight-bearing stepping, an effect that enhances rehabilitative training [201]. These data help clarify how human 5HT-secreting neuronal grafts might differently affect sensory and motor recovery after SCI based on graft location, since presumably, cell-based 5HT-secretion provided by either intrathecal or intraspinal hNT2.19 transplants, would be greatest nearest the lumbar dorsal horn, or ventral motor neurons, respectively.

## 5. Conclusions

To summarize the conclusions from 20 years of cell therapy studies, some comments and predictions can be made. (1) It is likely that only human cells will be useful as a source, whether primary tissue or cell lines, given that such sources are the least likely to be rejected by the host. Encapsulation technologies could be helpful here, if these technologies could keep the grafts viable and functional; (2) it is likely, at least for now, that only the simplest approaches to creating cell sources will be quickly approved for clinical trials, that is, not overly manipulated (in cell culture) or bioengineered cells (containing viral vectors); (3) some type of immunosuppression will be required, even for autologous sources, but such regimens could be tested rigorously in pre-clinical experiments, that is, nonhuman primates; (4) an intrathecal graft site would likely be the best for cell injections for the treatment of neuropathic pain. Any other transplant type would need to be placed as near to its “site-of-action” as is reasonable, especially if grafted cells are known to not migrate, such as with NT2 cells; (5) if cells are used for anti-nociception, and placed intrathecally, those that passively secrete inhibitory (or drive inhibitory systems) neurotransmitters would likely work the best, rather than cells that secrete any number of known and unknown agents; (6) transplant sources need to be tested in as many pre-clinical peripheral and central models of motor and sensory injury as possible, to avoid later, and inappropriate, “off-label” use/side-effects in humans; (7) a pragmatic, rather than a purely mechanistic, approach can be used for pre-clinical work. It is more useful that cell therapy approaches are tested, without necessarily understanding how they work, as long as such technologies are proven as safe as possible; (8) all efforts should be taken to keep patients/provider costs as low as possible, so that cell therapy can be applied almost as readily as pharmacologic treatments; (9) the rapid establishment of a Research Ethics Consortium should be established, to be tasked to assemble an interdisciplinary panel of experts who will apply ethical principles to analyze the social merit relative to the economic incentives of this emerging technology [207]. Any consortium would evaluate how these novel ethical issues in emerging technologies are addressed under current oversight and regulatory structures and where there may be gaps and need for revised or new public policy approaches.

Our own data, and recent data from others [66, 67, 84, 207], demonstrate that highly effective, safe, and reproducible delivery of potential cell therapeutic candidates into brain and spinal sites can be achieved across a wide range of cell doses by either intrathecal or direct parenchymal injections. The hNT2 or hNT2-derived cell lines, such as hNT2.17 and hNT2.19, have great potential to permanently reverse symptoms of neuropathic pain following PNS and CNS injuries and can offer new hope to treat these intractable conditions to significantly improve human health.
